# Supplementary material for: Black Grouse Hissing Calls Show Geographic Variability across the Distribution Area
Source: Animals (Basel). 2023 Jun 1;13(11):1844. doi: 10.3390/ani13111844 (PMC10251829; doi:10.3390/ani13111844)
Supplement: Supplementary file 1 [file animals-13-01844-s001.zip › Supplemental Table S1.pdf]

Country	ID	High Freq (Hz)	Agg Entropy (bits)	Delta Time (s)	Dur 50% (s)	Freq 25% (Hz)	Freq 5% (Hz)	Freq 95% (Hz)	Min Entropy (bits)	Time 25% Rel,	Time 5% Rel,	Time 95% (s)	Time 95% Rel,
Czech Republic	1	1664,2	2,338	0,7312	0,2560	1031,25	843,75	1312,50	1,332	0,1103	0,0809	60,6773	0,7941
Czech Republic	1	1751,8	2,380	0,8578	0,2293	1125,00	750,00	1312,50	1,420	0,1437	0,0750	62,6133	0,7375
Czech Republic	1	1751,8	2,596	0,8156	0,2133	1031,25	562,50	1312,50	1,468	0,1974	0,0987	64,2080	0,7829
Czech Republic	1	1839,4	2,932	0,8999	0,3840	1031,25	750,00	1500,00	1,632	0,2798	0,1012	78,5493	0,9107
Czech Republic	1	1751,8	2,122	0,7734	0,2667	1125,00	1031,25	1312,50	1,333	0,1389	0,0625	80,8213	0,7778
Czech Republic	1	1751,8	2,147	0,7734	0,3573	1125,00	1031,25	1312,50	1,295	0,1389	0,0903	84,6453	0,8333
Czech Republic	2	1927,0	3,165	0,8737	0,4800	1125,00	562,50	1593,75	1,127	0,1288	0,0675	10,7947	0,9080
Czech Republic	2	2014,6	2,727	1,2759	0,2880	1312,50	1031,25	1593,75	1,326	0,4874	0,0756	44,5867	0,8403
Czech Republic	2	2189,8	3,112	1,1650	0,3413	1218,75	656,25	1593,75	1,565	0,4516	0,1198	53,1733	0,8986
Czech Republic	2	2102,2	3,497	1,0402	0,3253	1218,75	562,50	1687,50	1,532	0,5000	0,1289	60,2987	0,9124
Czech Republic	2	2102,2	2,348	1,1095	0,1707	1312,50	1031,25	1500,00	1,317	0,5652	0,1836	72,1600	0,8599
Czech Republic	2	2014,6	2,765	1,1095	0,2400	1312,50	656,25	1500,00	1,283	0,4686	0,1014	80,4640	0,8502
Czech Republic	2	2014,6	2,859	0,9847	0,1653	1312,50	656,25	1500,00	1,435	0,5792	0,1257	108,8853	0,8634
Czech Republic	3	2108,5	2,315	1,1691	0,1973	1312,50	1125,00	1593,75	1,290	0,4954	0,1789	11,0880	0,8257
Czech Republic	3	2170,5	3,066	1,4556	0,2347	1312,50	1031,25	1875,00	1,399	0,5441	0,1324	20,8213	0,8235
Czech Republic	3	2294,6	3,313	1,0659	0,3680	1312,50	1125,00	2062,50	1,390	0,3939	0,0707	24,5813	0,8889
Czech Republic	3	2294,6	2,676	0,9399	0,3467	1218,75	1125,00	1781,25	1,354	0,0914	0,0629	27,5733	0,8514
Czech Republic	3	2108,5	2,978	0,9399	0,2293	1312,50	750,00	1593,75	1,410	0,5600	0,1143	52,2027	0,9429
Czech Republic	4	2506,5	2,524	1,1268	0,0960	1406,25	1312,50	1781,25	1,392	0,6019	0,0995	5,9467	0,7725
Czech Republic	4	2381,2	2,456	1,0321	0,0800	1218,75	843,75	1500,00	0,758	0,1347	0,0725	14,3307	0,7150
Czech Republic	4	2255,9	2,762	1,0426	0,5440	1312,50	1125,00	1593,75	1,252	0,1598	0,0722	20,8267	0,8454
Czech Republic	4	2443,9	3,152	1,1058	0,6293	1312,50	1125,00	1875,00	1,338	0,1019	0,0631	28,9653	0,8786
Czech Republic	4	2631,9	2,728	1,0742	0,5600	1312,50	1125,00	1687,50	1,264	0,1150	0,0800	47,7547	0,8200
Czech Republic	4	2318,5	2,755	0,9267	0,2507	1312,50	1125,00	1593,75	1,288	0,4855	0,1040	75,8507	0,8497
Czech Republic	4	2193,2	2,651	0,9899	0,7733	1406,25	1218,75	1781,25	1,383	0,0595	0,0432	80,0907	0,9514
Czech Republic	4	2318,5	2,557	1,2216	0,2933	1406,25	1218,75	1687,50	1,307	0,4693	0,0614	159,1680	0,8289
Czech Republic	4	2255,9	2,492	1,2006	0,6240	1312,50	1218,75	1593,75	1,298	0,1027	0,0759	169,7067	0,7277
Czech Republic	4	2506,5	2,704	1,1268	0,5813	1406,25	1218,75	1687,50	1,415	0,1190	0,0714	171,0987	0,7571

Czech Republic	4	2005,2	2,665	0,9794	0,5973	1312,50	1125,00	1593,75	1,251	0,1484	0,0604	191,7173	0,8681
Czech Republic	4	2193,2	2,782	1,0847	0,6400	1406,25	1218,75	1781,25	1,492	0,1330	0,0739	223,4933	0,7931
Czech Republic	4	2381,2	2,654	0,8952	0,5707	1312,50	1125,00	1593,75	1,452	0,1377	0,0659	259,5840	0,8802
Czech Republic	5	2711,5	3,892	0,9388	0,5653	1312,50	843,75	2062,50	1,609	0,1257	0,0743	16,6453	0,8229
Czech Republic	5	2596,2	3,523	1,0078	0,6027	1312,50	1125,00	1968,75	1,608	0,1064	0,0798	58,2080	0,8404
Czech Republic	5	2365,4	3,001	1,0492	0,5760	1406,25	1218,75	1875,00	1,415	0,1333	0,0564	123,0187	0,8256
Czech Republic	5	2365,4	2,978	0,8283	0,5653	1312,50	1218,75	1781,25	1,435	0,0779	0,0584	133,3707	0,9091
Czech Republic	5	2365,4	3,150	1,0492	0,6027	1312,50	1125,00	1875,00	1,361	0,1480	0,0714	148,2080	0,8520
Czech Republic	5	2538,5	3,013	0,9250	0,5547	1312,50	1125,00	1781,25	1,619	0,0988	0,0756	154,7787	0,8372
Czech Republic	5	2711,5	3,386	1,2563	0,4320	1593,75	1125,00	2062,50	1,333	0,4231	0,0641	164,0373	0,8974
Czech Republic	5	2480,8	3,474	0,8698	0,4800	1406,25	937,50	1968,75	1,738	0,1358	0,0864	199,1520	0,8642
Czech Republic	5	2538,5	3,277	1,0492	0,6133	1500,00	937,50	1968,75	1,205	0,1020	0,0765	201,3440	0,8316
Czech Republic	5	2423,1	2,431	1,1321	0,2080	1312,50	1218,75	1593,75	1,322	0,5403	0,0900	208,2400	0,8152
Czech Republic	5	2423,1	3,074	1,0078	0,2400	1312,50	1218,75	1875,00	1,353	0,5053	0,0638	220,8587	0,8830
Czech Republic	5	2596,2	3,461	1,0078	0,5813	1500,00	1218,75	2062,50	1,046	0,0904	0,0638	228,1600	0,8085
Czech Republic	6	2713,0	2,715	1,3315	0,2027	1500,00	1406,25	1875,00	1,398	0,5743	0,0964	28,0213	0,8233
Czech Republic	6	2504,3	3,070	1,2427	0,6987	1406,25	1312,50	1875,00	1,288	0,0948	0,0647	33,1147	0,7974
Czech Republic	6	2991,3	3,297	1,0398	0,6560	1312,50	1218,75	1875,00	1,599	0,1082	0,0722	40,4533	0,8402
Czech Republic	6	2643,5	2,954	1,1920	0,5440	1500,00	1312,50	1875,00	0,769	0,1126	0,0901	46,7253	0,7387
Czech Republic	6	2504,3	3,236	1,0779	0,5600	1406,25	1218,75	1968,75	0,811	0,1040	0,0644	50,9227	0,7673
Czech Republic	6	2504,3	3,260	1,0652	0,6133	1406,25	1218,75	1968,75	1,706	0,1106	0,0704	57,4027	0,7688
Czech Republic	6	2713,0	3,227	0,8876	0,4853	1312,50	1218,75	1875,00	1,719	0,1091	0,0788	78,9440	0,8242
Czech Republic	6	2573,9	3,246	1,0271	0,6027	1312,50	843,75	1781,25	0,949	0,1094	0,0729	114,7787	0,8958
Czech Republic	6	2504,3	2,822	0,9003	0,4853	1406,25	1312,50	1781,25	1,478	0,0958	0,0599	154,8960	0,7305
Czech Republic	6	2365,2	2,877	0,9637	0,5280	1312,50	1218,75	1687,50	1,272	0,0950	0,0670	163,0187	0,8212
Czech Republic	6	2504,3	2,616	1,1413	0,1707	1406,25	1218,75	1687,50	1,280	0,4789	0,1221	171,7547	0,7465
Czech Republic	6	2643,5	3,097	0,9003	0,5813	1500,00	1312,50	1968,75	1,408	0,0833	0,0595	178,1120	0,8393
Czech Republic	6	2156,5	3,065	1,2934	0,7573	1218,75	1125,00	1781,25	1,235	0,0992	0,0620	249,9040	0,8017
Czech Republic	6	2504,3	3,166	1,3315	0,2880	1406,25	1218,75	1875,00	1,449	0,4355	0,0645	286,8267	0,7863
Czech Republic	6	2504,3	3,190	1,2427	0,6773	1406,25	1125,00	1875,00	1,211	0,0948	0,0647	291,5147	0,8190
Czech Republic	6	2434,8	3,386	1,1286	0,6133	1218,75	1125,00	1875,00	1,346	0,1381	0,0857	293,4293	0,8333

Czech Republic	6	2643,5	3,245	1,1159	0,6720	1500,00	1312,50	1968,75	1,513	0,1106	0,0817	295,8347	0,8173
Czech Republic	6	2226,1	2,876	1,2807	0,3467	1312,50	1125,00	1687,50	1,460	0,4500	0,0625	310,8053	0,8083
Czech Republic	7	2497,1	2,816	0,9071	0,5333	1406,25	1218,75	1781,25	1,481	0,1479	0,0888	28,7307	0,8994
Czech Republic	7	2427,7	2,467	0,9761	0,5440	1406,25	1312,50	1781,25	1,334	0,1374	0,0934	42,3467	0,8626
Czech Republic	7	2566,5	2,539	0,8774	0,4587	1406,25	1218,75	1687,50	1,333	0,1350	0,0859	59,0400	0,8896
Czech Republic	7	2289,0	2,952	1,0707	0,5813	1406,25	1218,75	1875,00	1,074	0,1500	0,0850	80,7627	0,8550
Czech Republic	7	2427,7	2,711	1,0855	0,6027	1406,25	1312,50	1781,25	1,363	0,2020	0,0887	92,1973	0,8867
Czech Republic	7	2497,1	2,790	1,0409	0,4427	1406,25	1312,50	1781,25	1,372	0,2769	0,0872	153,4347	0,8769
Czech Republic	7	2635,8	2,910	1,0707	0,4160	1406,25	1312,50	1875,00	1,258	0,3400	0,1000	180,0693	0,8550
Czech Republic	7	2358,4	2,915	1,0707	0,6027	1406,25	1312,50	1875,00	1,315	0,1307	0,0905	222,6880	0,8894
Czech Republic	7	2427,7	2,675	0,8922	0,4107	1406,25	1312,50	1781,25	1,286	0,1084	0,0723	240,7467	0,8253
Czech Republic	7	2289,0	2,598	0,8922	0,4640	1406,25	1312,50	1687,50	1,358	0,1687	0,1205	261,6213	0,8675
Czech Republic	7	2289,0	2,775	0,9814	0,4960	1406,25	1218,75	1875,00	1,330	0,1858	0,0656	318,3147	0,8852
Czech Republic	8	2176,2	2,808	0,9837	0,4800	1406,25	1218,75	1781,25	1,377	0,1803	0,0656	11,6587	0,8525
Czech Republic	8	2362,7	3,078	1,0274	0,2773	1406,25	1218,75	2156,25	1,354	0,4375	0,0990	47,8347	0,8125
Czech Republic	8	2176,2	2,375	0,8526	0,5173	1406,25	1218,75	1687,50	1,427	0,1132	0,0629	63,6587	0,8302
Czech Republic	8	2176,2	2,805	0,7870	0,4427	1406,25	1218,75	1781,25	1,460	0,0952	0,0476	92,0907	0,8095
Czech Republic	8	2627,7	2,860	0,9093	0,4213	1406,25	1218,75	1968,75	1,324	0,0888	0,0592	13,9413	0,7456
Czech Republic	8	2189,8	2,262	0,7558	0,4213	1406,25	1312,50	1687,50	1,437	0,0786	0,0500	19,7653	0,8000
Czech Republic	8	2189,8	2,535	0,9329	0,1600	1406,25	1218,75	1687,50	1,391	0,5172	0,0805	44,9013	0,7931
Czech Republic	8	2102,2	2,971	0,8384	0,4587	1406,25	1218,75	1781,25	1,636	0,0892	0,0573	144,3787	0,8280
Czech Republic	9	2277,4	2,604	0,7088	0,1813	1500,00	1218,75	1687,50	1,406	0,4697	0,0909	34,4427	0,8258
Czech Republic	9	2540,1	2,647	0,8544	0,2773	1406,25	1312,50	2250,00	1,366	0,3019	0,0818	38,7787	0,7610
Czech Republic	9	2102,2	2,608	0,8861	0,5493	1500,00	1218,75	1687,50	1,281	0,1758	0,0727	65,1947	0,8667
Czech Republic	9	2802,9	2,318	0,8987	0,4587	1406,25	1312,50	1687,50	1,291	0,1557	0,0719	77,5467	0,8084
Czech Republic	9	2452,6	2,591	1,0374	0,5120	1312,50	1218,75	1593,75	1,289	0,1140	0,0570	15,5787	0,7824
Czech Republic	9	2627,7	2,942	1,0522	0,4160	1406,25	1312,50	1875,00	1,245	0,2194	0,0867	39,6000	0,8316
Czech Republic	9	2540,1	2,522	1,0522	0,2027	1500,00	1312,50	1781,25	1,263	0,5408	0,1122	90,8587	0,8673
Czech Republic	10	2365,0	3,237	0,8594	0,1973	1593,75	1218,75	2062,50	1,458	0,6625	0,1000	8,6720	0,9563
Czech Republic	10	2627,7	3,056	0,8254	0,3787	1406,25	1218,75	1968,75	1,353	0,1429	0,0714	12,2400	0,7532
Czech Republic	10	2715,3	3,553	0,8368	0,3573	1500,00	1218,75	2156,25	1,263	0,3910	0,0641	113,0240	0,9295

Czech Republic	10	2540,1	3,158	0,7802	0,4587	1406,25	1218,75	1968,75	1,310	0,1379	0,1034	201,6373	0,8207
Czech Republic	10	2627,7	2,855	0,7865	0,3680	1312,50	1218,75	1781,25	1,332	0,1088	0,0748	105,4613	0,7211
Czech Republic	10	2365,0	2,766	0,7770	0,3680	1406,25	1218,75	1781,25	1,343	0,0966	0,0690	116,6933	0,6621
Czech Republic	10	2540,1	3,217	0,9965	0,5387	1500,00	1312,50	2062,50	1,413	0,1129	0,0860	23,0827	0,7957
Czech Republic	10	2452,6	2,930	0,8521	0,4267	1406,25	1218,75	1968,75	1,292	0,0881	0,0629	81,2053	0,8176
Czech Republic	11	2102,2	2,482	1,0170	0,6027	1500,00	1312,50	1781,25	1,343	0,0847	0,0582	33,4240	0,8042
Czech Republic	11	2452,6	2,962	0,9055	0,4747	1500,00	1406,25	1968,75	1,323	0,1420	0,0710	65,1147	0,8047
Czech Republic	11	2540,1	3,809	1,0309	0,2880	1312,50	1031,25	2437,50	1,950	0,4508	0,0622	73,8667	0,8705
Czech Republic	11	2452,6	3,122	0,9752	0,4907	1500,00	1312,50	2250,00	1,520	0,1593	0,0714	76,9067	0,8242
Czech Republic	11	2452,6	3,322	0,9334	0,5387	1406,25	1218,75	1968,75	1,721	0,1149	0,0747	80,1653	0,8621
Czech Republic	11	2715,3	3,128	1,1284	0,4213	1406,25	1125,00	1875,00	1,325	0,3839	0,0948	91,4560	0,8863
Czech Republic	11	3115,4	3,654	1,1220	0,2453	1406,25	1312,50	2250,00	1,645	0,5048	0,0952	27,2213	0,8857
Czech Republic	11	2192,3	3,112	0,8144	0,3627	1312,50	1218,75	1781,25	1,668	0,2039	0,1579	28,5760	0,7500
Czech Republic	11	2538,5	3,456	0,8325	0,4160	1406,25	1218,75	2250,00	1,775	0,1806	0,1161	30,7413	0,8581
Czech Republic	11	2653,8	3,582	1,0677	0,5173	1218,75	937,50	1968,75	1,015	0,1910	0,1005	62,9867	0,8593
Czech Republic	11	2538,5	3,359	1,0677	0,2613	1406,25	1218,75	1968,75	1,723	0,4673	0,0603	81,5200	0,8543
Czech Republic	12	2537,9	2,448	1,1033	0,3653	1125,00	562,50	1687,50	0,414	0,2906	0,0678	20,6000	0,8111
Czech Republic	12	2427,6	2,365	0,8874	0,2987	1125,00	750,00	1687,50	1,042	0,2054	0,0876	97,8293	0,7825
Czech Republic	12	2537,9	2,518	0,9713	0,2560	1125,00	937,50	1687,50	1,400	0,2912	0,0989	101,3653	0,7363
Czech Republic	12	2758,6	2,465	1,0793	0,4480	1312,50	937,50	1875,00	1,391	0,2252	0,1015	106,2453	0,7847
Czech Republic	12	2758,6	2,290	1,2232	0,5920	1125,00	937,50	1687,50	1,396	0,1138	0,0613	111,6720	0,8118
Czech Republic	12	2427,6	2,184	1,2112	0,2240	1125,00	937,50	1687,50	1,260	0,2936	0,0883	143,6587	0,7417
Czech Republic	12	2096,6	2,479	1,2112	0,3840	1125,00	937,50	1687,50	1,286	0,2826	0,0773	170,5973	0,7638
Czech Republic	12	2447,6	2,103	1,1022	0,1440	1125,00	937,50	1687,50	1,332	0,4587	0,1117	36,4400	0,7937
Czech Republic	12	2447,6	2,257	1,0555	0,2347	1125,00	937,50	1687,50	1,253	0,2937	0,1114	40,8027	0,7924
Czech Republic	12	2583,6	2,036	1,1022	0,3333	1125,00	937,50	1500,00	0,997	0,1262	0,0728	105,1120	0,6772
Czech Republic	12	2583,6	2,394	1,1769	0,4480	1125,00	187,50	1500,00	0,217	0,2114	0,0932	127,9333	0,7864
Czech Republic	12	2063,0	2,256	1,0149	0,3813	1125,00	937,50	1687,50	1,228	0,2190	0,0765	44,3333	0,7335
Czech Republic	12	2200,6	2,576	1,1455	0,3680	1125,00	187,50	1687,50	0,183	0,2313	0,0935	49,0933	0,8224
Czech Republic	12	2750,7	2,665	1,2460	0,3253	187,50	0,00	1500,00	0,723	0,2597	0,0665	107,0267	0,8305
Czech Republic	13	1975,4	3,286	1,0024	0,3657	947,46	861,33	1550,39	0,668	0,3509	0,0994	36,0606	0,9181

Czech Republic	13	1868,6	3,257	0,9241	0,3309	1033,59	775,20	1550,39	1,167	0,2722	0,1013	58,4969	0,8671
Czech Republic	13	1927,1	3,219	1,1763	0,5399	1291,99	947,46	1722,66	1,491	0,3069	0,0891	7,2736	0,9010
Czech Republic	13	1927,1	3,298	1,0359	0,5515	1119,73	775,20	1636,52	1,049	0,2022	0,1292	12,2659	0,8596
Czech Republic	13	1792,7	3,203	1,2115	0,4296	1033,59	861,33	1636,52	1,574	0,2899	0,0966	117,7135	0,8696
Czech Republic	13	1837,5	3,120	1,1588	0,3773	1119,73	1033,59	1722,66	1,091	0,2626	0,1212	124,9466	0,8333
Czech Republic	13	1792,7	3,289	1,2466	0,4238	1119,73	775,20	1636,52	1,010	0,3738	0,1262	142,7447	0,8832
Czech Republic	13	1703,0	2,660	0,9481	0,4470	1205,86	947,46	1464,26	1,277	0,1914	0,0926	145,8619	0,8210
Czech Republic	13	1523,8	2,979	1,1061	0,3309	1033,59	775,20	1378,13	1,392	0,2895	0,1526	195,9648	0,8579
Czech Republic	13	1882,3	3,406	1,2290	0,5050	689,06	602,93	1550,39	1,765	0,2844	0,0853	239,8447	0,9194
Czech Republic	13	1927,1	3,288	1,1763	0,4470	947,46	861,33	1636,52	1,045	0,2277	0,0842	247,8846	0,8564
Czech Republic	13	1972,0	3,245	1,1588	0,5457	947,46	861,33	1722,66	0,973	0,2663	0,1055	308,7615	0,8794
Czech Republic	14	2092,3	3,821	1,3173	0,5863	689,06	430,66	1464,26	1,762	0,3111	0,0667	25,1994	0,9333
Czech Republic	14	2172,8	2,929	1,2314	0,3019	1205,86	947,46	1550,39	1,122	0,3791	0,0758	38,8063	0,7773
Czech Republic	14	2011,9	3,661	1,1782	0,4122	775,20	602,93	1550,39	1,471	0,3218	0,0743	65,5673	0,8911
Czech Republic	14	2092,3	3,618	1,0996	0,5224	861,33	689,06	1722,66	1,849	0,2169	0,0741	77,2063	0,9259
Czech Republic	14	1931,4	3,438	1,1333	0,5573	861,33	689,06	1464,26	1,723	0,2103	0,0821	87,1851	0,8872
Czech Republic	15	2414,2	3,346	0,9599	0,2961	1291,99	1033,59	1722,66	1,768	0,4451	0,0976	35,6020	0,8354
Czech Republic	15	2253,3	3,150	1,0310	0,4470	1205,86	1033,59	1636,52	1,635	0,2655	0,1130	40,1125	0,8927
Czech Republic	15	2092,3	2,959	0,9421	0,4586	1119,73	947,46	1464,26	1,543	0,2174	0,0932	76,3530	0,8385
Czech Republic	15	2253,3	3,301	0,9776	0,3657	1119,73	861,33	1550,39	1,456	0,4012	0,0958	141,9610	0,8922
Czech Republic	15	2333,8	2,909	0,9421	0,2496	1119,73	947,46	1464,26	1,470	0,2360	0,1118	145,3163	0,7329
Czech Republic	15	1931,4	2,717	0,8888	0,3599	1205,86	947,46	1464,26	1,424	0,1842	0,1579	151,8759	0,7763
Czech Republic	16	1688,3	2,894	0,9595	0,5166	1033,59	861,33	1378,13	1,276	0,1524	0,0976	8,1966	0,9024
Czech Republic	16	1841,8	2,872	1,2130	0,4470	1119,73	861,33	1550,39	1,036	0,1250	0,1010	20,6077	0,7356
Czech Republic	16	1688,3	2,463	1,1044	0,1045	1205,86	947,46	1464,26	1,282	0,1323	0,1164	34,7777	0,7143
Czech Republic	16	1739,4	2,984	0,9595	0,4296	1033,59	775,20	1464,26	1,445	0,1402	0,0976	45,4356	0,8476
Czech Republic	16	1841,8	3,049	1,0138	0,3483	1033,59	775,20	1378,13	1,407	0,1329	0,0983	66,7109	0,8324
Czech Republic	16	1790,6	3,138	0,9776	0,4934	1033,59	775,20	1464,26	1,216	0,1617	0,0958	100,9720	0,8802
Czech Republic	16	1739,4	2,822	0,9957	0,4644	1119,73	861,33	1464,26	1,307	0,1520	0,1228	105,5753	0,8480
Czech Republic	16	1739,4	2,806	0,9595	0,4180	1119,73	775,20	1378,13	1,036	0,1402	0,1159	114,0448	0,8415
Czech Republic	16	1688,3	2,769	1,0863	0,4180	1119,73	775,20	1378,13	1,119	0,1290	0,1075	122,3924	0,7742

Czech Republic	16	1739,4	2,621	1,0319	0,5283	1119,73	1033,59	1464,26	1,458	0,1073	0,0734	130,8038	0,8362
Czech Republic	16	1637,1	3,069	1,1406	0,5050	1033,59	689,06	1464,26	1,397	0,1744	0,1077	141,3457	0,8718
Czech Republic	16	1637,1	3,100	0,9776	0,5108	775,20	689,06	1378,13	0,945	0,1437	0,0898	144,4978	0,9102
Czech Republic	17	2822,8	3,465	1,2634	0,2206	1722,66	1378,13	2411,72	1,549	0,5530	0,0876	13,9320	0,8203
Czech Republic	17	2343,5	3,573	1,2066	0,3889	1550,39	1291,99	2153,32	1,448	0,4348	0,0773	28,0671	0,8164
Czech Republic	17	2290,2	3,355	1,2208	0,2438	1636,52	1291,99	2067,19	1,476	0,5024	0,0861	36,6701	0,7847
Czech Republic	17	2237,0	3,102	1,1214	0,1974	1550,39	1291,99	1981,06	1,443	0,5233	0,1088	44,3443	0,7772
Czech Republic	17	2343,5	3,649	1,0646	0,4063	1378,13	1205,86	2067,19	1,943	0,1703	0,0659	48,7677	0,7747
Czech Republic	17	2343,5	3,452	1,0363	0,3019	1550,39	1378,13	2153,32	1,618	0,5254	0,0734	58,8161	0,8927
Czech Republic	17	2237,0	3,454	0,8375	0,3947	1378,13	1205,86	1981,06	1,746	0,1042	0,0694	69,9211	0,7431
Czech Republic	17	2609,8	3,551	1,1356	0,3657	1550,39	1205,86	2067,19	1,829	0,3897	0,0718	87,4812	0,8154
Czech Republic	17	2290,2	3,271	1,0930	0,2554	1464,26	1205,86	1894,92	1,578	0,5080	0,0749	93,7622	0,8182
Czech Republic	17	2503,3	3,636	1,2066	0,3541	1550,39	1291,99	2239,45	1,649	0,5121	0,0773	113,1102	0,8889
Czech Republic	17	2290,2	3,306	1,2634	0,2844	1636,52	1378,13	2153,32	1,484	0,5576	0,0922	137,0848	0,8802
Czech Republic	17	2343,5	3,251	1,2208	0,2206	1636,52	1378,13	2153,32	1,319	0,6048	0,1238	154,8132	0,8714
Czech Republic	17	2237,0	3,234	1,1782	0,3541	1464,26	1291,99	1981,06	1,561	0,3861	0,0594	159,6256	0,7921
Czech Republic	17	2396,7	3,401	1,2350	0,2554	1636,52	1378,13	2153,32	1,567	0,5498	0,0900	188,1281	0,8578
Czech Republic	18	2414,2	3,546	1,0890	0,4180	1205,86	861,33	1722,66	1,564	0,4032	0,0914	24,0152	0,9032
Czech Republic	18	2253,3	3,463	1,0527	0,3599	1291,99	1033,59	1808,79	1,661	0,4778	0,0722	47,6589	0,9111
Czech Republic	18	2655,7	3,523	1,2161	0,2961	1378,13	1119,73	1894,92	1,576	0,5529	0,1442	93,0365	0,8654
Czech Republic	18	2897,1	3,295	1,1253	0,6269	1291,99	1119,73	1808,79	1,395	0,1658	0,0984	110,0568	0,8342
Czech Republic	18	2414,2	3,262	1,0408	0,2902	1378,13	1119,73	1808,79	1,548	0,5506	0,1011	21,8558	0,8876
Czech Republic	19	1931,4	2,252	1,0251	0,2670	1119,73	1033,59	1378,13	1,298	0,1193	0,1023	23,8004	0,7500
Czech Republic	19	1770,4	2,095	0,9871	0,3715	1119,73	1033,59	1291,99	1,307	0,1775	0,0947	37,5989	0,7456
Czech Republic	19	1770,4	2,403	0,8100	0,3193	1119,73	1033,59	1378,13	1,319	0,1367	0,0791	74,0194	0,7122
Czech Republic	19	1609,5	2,685	0,9221	0,2844	1119,73	947,46	1464,26	1,524	0,2025	0,0886	45,3021	0,7468
Czech Republic	19	1770,4	2,222	1,1411	0,2728	1119,73	1033,59	1378,13	1,283	0,2398	0,1531	94,9290	0,6531
Czech Republic	19	1850,9	1,973	1,0604	0,1219	1119,73	1033,59	1378,13	1,290	0,1703	0,1154	99,2305	0,5110
Czech Republic	19	1690,0	2,511	0,9305	0,2380	1205,86	1033,59	1464,26	1,487	0,1824	0,0692	13,8043	0,7736
Czech Republic	19	1690,0	3,051	0,9305	0,2728	1119,73	602,93	1464,26	1,455	0,1572	0,0818	47,4674	0,8113
Czech Republic	20	2333,8	3,884	1,1927	0,3773	1205,86	1033,59	1981,06	1,859	0,3627	0,0931	15,9231	0,8431

Czech Republic	20	2253,3	3,796	1,0658	0,4934	1291,99	1033,59	2067,19	1,897	0,2088	0,0989	23,9978	0,8516
Czech Republic	20	2414,2	3,758	1,1419	0,5399	1291,99	689,06	1894,92	1,880	0,2041	0,1071	30,4297	0,8061
Czech Republic	20	2011,9	3,773	1,3703	0,4470	1119,73	947,46	1894,92	1,790	0,3191	0,1149	72,7423	0,7787
Czech Republic	20	2253,3	4,023	1,1927	0,5399	1205,86	775,20	2153,32	1,817	0,2108	0,1275	76,6142	0,7990
Czech Republic	20	2253,3	3,938	1,2434	0,4702	1205,86	1033,59	2067,19	1,923	0,3568	0,1315	105,3896	0,9108
Czech Republic	20	2092,3	3,958	1,1165	0,5108	1119,73	516,80	1894,92	2,036	0,2670	0,1152	124,7318	0,9005
Czech Republic	20	2172,8	3,902	1,3957	0,5805	1119,73	861,33	1981,06	1,789	0,1667	0,0875	160,0319	0,7708
Scotland	21	2579,7	3,203	0,9990	0,2240	1687,50	1312,50	2062,50	1,617	0,4545	0,0909	89,0453	0,8128
Scotland	21	2521,7	3,120	0,9701	0,4000	1593,75	1312,50	2062,50	1,366	0,1167	0,0833	92,1120	0,7167
Scotland	21	2260,9	3,246	0,9556	0,3680	1500,00	1218,75	1968,75	1,462	0,3202	0,0899	368,7413	0,8427
Scotland	21	2318,8	2,818	0,9266	0,5333	1500,00	1312,50	1875,00	1,419	0,0751	0,0289	371,0987	0,7861
Scotland	21	2541,5	3,227	1,0622	0,6187	1593,75	1312,50	2062,50	1,779	0,0960	0,0707	55,8933	0,8636
Scotland	21	2664,5	3,271	1,1803	0,6293	1593,75	1312,50	2062,50	1,675	0,1273	0,0636	169,9573	0,8364
Scotland	21	2582,5	3,426	1,0454	0,2560	1593,75	1218,75	2156,25	1,386	0,4872	0,0923	176,5280	0,8564
Scotland	21	2664,5	3,355	0,9611	0,5653	1687,50	1406,25	2156,25	1,702	0,1173	0,0726	424,8213	0,7989
Scotland	21	2500,5	3,259	1,0319	0,5973	1593,75	1406,25	2062,50	1,403	0,1042	0,0521	68,7040	0,8177
Scotland	21	2377,5	3,063	0,9429	0,5067	1593,75	1406,25	2062,50	1,347	0,1257	0,0857	258,9280	0,7943
Scotland	21	2295,5	3,354	0,8896	0,2667	1500,00	1312,50	2062,50	1,615	0,5663	0,0904	299,2853	0,9398
Scotland	21	2418,5	3,176	0,9459	0,5813	1687,50	1406,25	2156,25	1,570	0,1023	0,0682	67,7387	0,8580
Scotland	21	2459,5	3,213	0,9291	0,5227	1593,75	1500,00	2156,25	1,471	0,1098	0,0636	134,7147	0,8092
Scotland	22	3238,3	4,121	0,9558	0,3733	1968,75	1593,75	3000,00	2,290	0,0730	0,0506	53,6960	0,8539
Scotland	22	3402,3	4,532	0,9440	0,5653	1687,50	1218,75	3281,25	2,394	0,1591	0,0625	260,0320	0,9091
Scotland	22	3217,4	3,762	1,0004	0,4800	1593,75	1218,75	2437,50	0,926	0,0588	0,0160	26,5760	0,8128
Scotland	22	2910,4	3,610	1,0627	0,5653	1875,00	1593,75	2531,25	1,816	0,1364	0,0707	1168,6453	0,8788
Scotland	22	2500,5	3,254	0,8784	0,4267	1687,50	1500,00	2250,00	1,608	0,1098	0,0671	1175,3280	0,8171
Scotland	22	3115,4	3,710	1,0085	0,4267	1593,75	1406,25	2531,25	1,752	0,1170	0,0585	1199,2160	0,7340
Scotland	22	3156,4	3,862	0,9326	0,5333	1781,25	1500,00	2718,75	1,938	0,0632	0,0460	1388,2560	0,8448
Scotland	23	2828,4	3,837	0,9355	0,5760	1687,50	1406,25	2531,25	2,068	0,0800	0,0343	214,9173	0,8457
Scotland	23	2746,4	3,916	0,8665	0,5333	1593,75	1312,50	2531,25	2,297	0,0807	0,0373	223,3227	0,8634
Scotland	23	2541,5	3,917	0,9125	0,5760	1593,75	1031,25	2343,75	1,999	0,1000	0,0471	282,9440	0,8765
Scotland	23	2213,6	3,624	0,8665	0,5387	1406,25	1125,00	2156,25	1,897	0,0994	0,0373	335,9680	0,8944

Scotland	23	2459,5	3,913	0,9202	0,5867	1406,25	1031,25	2343,75	2,056	0,1170	0,0585	342,3040	0,9298
Scotland	23	2787,4	3,847	0,8895	0,5920	1593,75	1312,50	2531,25	1,598	0,0788	0,0485	368,9973	0,9030
Scotland	23	2623,5	3,914	1,0735	0,3307	1593,75	1125,00	2343,75	1,891	0,4450	0,0550	381,0827	0,9050
Scotland	23	2377,5	3,738	1,1603	0,3200	1500,00	843,75	2156,25	1,728	0,4630	0,0370	917,1733	0,8935
Scotland	23	2336,5	3,409	1,0844	0,3093	1687,50	1312,50	2156,25	1,833	0,4384	0,0345	1063,3600	0,8522
Scotland	23	2418,5	3,366	1,0736	0,5333	1687,50	1500,00	2343,75	1,796	0,1000	0,0550	1223,5093	0,7250
Scotland	23	2828,4	3,843	1,0302	0,3040	1968,75	1593,75	2812,50	1,996	0,4479	0,0729	1234,0373	0,8854
Scotland	23	2582,5	3,824	1,0953	0,2773	1781,25	1218,75	2531,25	2,122	0,4853	0,0392	1239,2373	0,8775
Scotland	23	2608,7	3,542	1,4109	0,3360	1781,25	1500,00	2343,75	1,960	0,5492	0,0682	1390,0960	0,9015
Scotland	24	2582,5	3,507	1,2146	0,6987	1593,75	1406,25	2343,75	1,782	0,1239	0,0619	408,7573	0,8584
Scotland	24	2459,5	3,324	1,1603	0,6987	1687,50	1406,25	2156,25	1,710	0,0968	0,0553	433,4560	0,8664
Scotland	24	2459,5	3,421	1,0194	0,5813	1593,75	1406,25	2250,00	1,849	0,1204	0,0733	449,4987	0,8848
Scotland	24	2500,5	3,507	0,9109	0,5813	1593,75	1312,50	2250,00	1,813	0,1000	0,0353	459,6960	0,9059
Scotland	24	2418,5	3,382	1,0519	0,5547	1687,50	1312,50	2250,00	1,670	0,0612	0,0357	552,1067	0,8827
Scotland	24	2459,5	3,378	1,0085	0,5600	1593,75	1406,25	2156,25	1,519	0,0851	0,0479	644,5920	0,8777
Scotland	24	2500,5	3,254	0,9977	0,4960	1593,75	1406,25	2156,25	1,656	0,0914	0,0591	657,4133	0,8387
Scotland	24	2705,5	3,560	1,1820	0,7253	1500,00	1312,50	2437,50	1,536	0,0950	0,0407	715,0773	0,9005
Scotland	24	2459,5	3,136	1,0194	0,4480	1500,00	1406,25	2250,00	1,380	0,0895	0,0421	717,6427	0,8737
Scotland	24	2582,5	3,515	1,2363	0,7733	1593,75	1312,50	2343,75	1,781	0,0823	0,0476	764,1600	0,8788
Scotland	24	2623,5	3,247	1,1061	0,7253	1593,75	1406,25	2156,25	1,690	0,1117	0,0340	902,4853	0,9029
Scotland	24	2664,5	2,829	1,2688	0,2613	1781,25	1593,75	2156,25	1,412	0,5781	0,0549	1002,6453	0,8776
Scotland	24	2664,5	3,241	1,2363	0,7307	1593,75	1406,25	2156,25	1,703	0,0519	0,0346	1005,2267	0,8139
Scotland	24	2336,5	3,163	1,0411	0,6187	1593,75	1406,25	2062,50	1,602	0,0923	0,0564	1061,5040	0,8462
Scotland	24	2582,5	3,109	1,2037	0,2347	1593,75	1406,25	2062,50	1,382	0,5689	0,0711	1139,0080	0,8356
Scotland	24	2664,5	3,185	1,0085	0,6347	1593,75	1406,25	2156,25	1,563	0,0745	0,0319	1144,7733	0,8564
Scotland	24	2705,5	3,047	1,1820	0,2773	1781,25	1593,75	2250,00	1,550	0,5430	0,0633	1182,1227	0,8869
Scotland	24	2746,4	3,046	1,2363	0,3627	1781,25	1593,75	2250,00	1,525	0,4416	0,0563	1226,6293	0,8615
Scotland	24	2377,5	3,012	1,0085	0,6027	1593,75	1406,25	2062,50	1,439	0,0691	0,0372	1228,4747	0,8085
Scotland	24	2295,5	3,080	0,9326	0,3200	1500,00	1312,50	2062,50	1,512	0,0805	0,0575	1230,5653	0,7586
Scotland	24	2582,5	3,092	1,1603	0,2773	1781,25	1593,75	2250,00	1,428	0,4722	0,0370	1254,4053	0,8519
Scotland	24	2623,5	3,103	1,1820	0,4160	1687,50	1500,00	2250,00	1,696	0,3937	0,0452	1300,6027	0,8824

Scotland	24	2459,5	3,131	1,0519	0,6720	1687,50	1500,00	2156,25	1,452	0,1020	0,0663	1307,5840	0,9082
Scotland	24	2787,4	3,314	1,2254	0,3573	1687,50	1500,00	2250,00	1,669	0,4236	0,0568	1348,6933	0,8035
Scotland	24	2746,4	3,211	1,0519	0,6507	1593,75	1406,25	2156,25	1,487	0,0765	0,0459	1352,7947	0,8163
Scotland	25	2500,5	3,562	1,1812	0,3040	1593,75	1312,50	2343,75	1,890	0,5068	0,0724	238,9760	0,9050
Scotland	25	2377,5	3,375	0,9886	0,5333	1500,00	1312,50	2156,25	1,733	0,1957	0,0598	325,1947	0,8641
Scotland	25	2254,5	3,251	0,6933	0,3093	1500,00	1312,50	2062,50	1,952	0,1318	0,0775	601,1040	0,7442
Scotland	25	2336,5	3,225	0,9758	0,2133	1593,75	1312,50	2062,50	1,661	0,4560	0,0879	611,1627	0,7802
Scotland	25	2459,5	3,406	1,1041	0,3947	1500,00	1312,50	2156,25	1,649	0,2621	0,0388	616,7253	0,7864
Scotland	25	2500,5	3,397	0,9886	0,4107	1500,00	1312,50	2156,25	1,520	0,2865	0,0865	629,3013	0,8324
Scotland	25	2500,5	3,362	1,0271	0,5120	1500,00	1218,75	2156,25	1,473	0,1562	0,0521	674,4800	0,8073
Scotland	25	2459,5	3,390	1,0656	0,3253	1500,00	1218,75	2062,50	1,638	0,2965	0,0503	677,6373	0,7387
Scotland	25	2418,5	3,397	0,9372	0,4533	1500,00	1312,50	2156,25	1,618	0,1379	0,0517	685,8613	0,7816
Scotland	25	2459,5	3,631	1,2599	0,4373	1593,75	1218,75	2250,00	1,851	0,4170	0,0511	189,0240	0,8553
Scotland	25	2582,5	3,640	1,1851	0,4693	1500,00	1218,75	2156,25	1,766	0,2613	0,0360	195,6907	0,7928
Scotland	25	2377,5	3,621	1,2385	0,4213	1593,75	1218,75	2156,25	1,703	0,3333	0,0519	252,4053	0,8139
Scotland	25	2377,5	3,316	1,1638	0,5120	1500,00	1218,75	2062,50	0,921	0,0596	0,0459	430,6187	0,7294
Scotland	25	2500,5	3,652	1,2919	0,4480	1500,00	1218,75	2156,25	1,623	0,3760	0,0579	579,5733	0,8388
Scotland	26	2377,5	3,085	0,9886	0,2400	1500,00	1406,25	2062,50	1,600	0,0543	0,0217	612,7520	0,7337
Scotland	26	2418,5	3,166	1,1298	0,6080	1406,25	1218,75	1968,75	1,465	0,0900	0,0474	614,9867	0,8246
Scotland	26	2418,5	3,464	1,1555	0,5707	1406,25	1125,00	1968,75	1,676	0,1065	0,0278	626,0747	0,8611
Scotland	26	2500,5	3,435	1,1170	0,5813	1406,25	1218,75	1968,75	1,811	0,1394	0,0385	630,8053	0,8606
Scotland	26	2377,5	3,580	1,1427	0,5227	1312,50	1125,00	2062,50	1,497	0,0892	0,0469	641,7227	0,8169
Scotland	26	2459,5	3,572	1,1555	0,4373	1406,25	1218,75	2156,25	1,775	0,0741	0,0463	658,9120	0,8009
Scotland	27	2105,3	2,629	0,9396	0,5440	1406,25	1218,75	1687,50	1,506	0,1657	0,0629	180,5547	0,8343
Scotland	27	2263,2	2,794	1,0143	0,5813	1406,25	1312,50	1781,25	1,360	0,1746	0,0635	186,4320	0,8677
Scotland	27	2157,9	2,613	1,1745	0,3413	1406,25	1218,75	1687,50	1,491	0,3881	0,0457	229,1307	0,7991
Scotland	27	2368,4	2,602	1,1211	0,3093	1406,25	1312,50	1687,50	1,405	0,5571	0,0667	284,8747	0,9143
Scotland	27	2157,9	2,515	1,1424	0,3093	1406,25	1218,75	1687,50	1,472	0,4366	0,0329	292,0800	0,8263
Scotland	27	2210,5	2,683	1,0357	0,2720	1406,25	1312,50	1781,25	1,430	0,4041	0,0363	304,9973	0,8135
Scotland	27	2263,2	2,700	1,1745	0,2933	1406,25	1218,75	1781,25	1,420	0,5251	0,0731	313,8773	0,8858
Scotland	27	2315,8	2,647	1,2278	0,2880	1406,25	1312,50	1781,25	1,401	0,5546	0,0786	373,4453	0,8821

Scotland	27	2526,3	2,756	1,2492	0,2667	1500,00	1312,50	1781,25	1,475	0,5579	0,1073	433,7333	0,8627
Scotland	27	2315,8	3,511	1,0784	0,7627	1406,25	1125,00	2062,50	0,718	0,0697	0,0448	437,9627	0,9403
Scotland	27	2368,4	2,826	1,1638	0,3360	1406,25	1312,50	1781,25	1,478	0,3825	0,0553	441,4240	0,8295
Scotland	27	2315,8	2,854	1,2492	0,3947	1406,25	1218,75	1781,25	1,351	0,4206	0,0601	483,6587	0,8755
Scotland	27	2157,9	2,677	1,2599	0,3413	1406,25	1218,75	1781,25	1,475	0,4746	0,0551	564,6187	0,8686
Scotland	27	2315,8	2,642	1,0997	0,2720	1406,25	1312,50	1781,25	1,338	0,5437	0,0680	576,7147	0,9126
Scotland	27	2263,2	2,746	1,2065	0,3787	1406,25	1312,50	1781,25	1,381	0,5378	0,0711	607,0560	0,9200
Scotland	28	3361,3	4,114	1,0647	0,2720	1968,75	1593,75	3000,00	1,650	0,4774	0,0704	459,2213	0,8794
Scotland	28	3074,4	4,161	1,0005	0,3840	1593,75	1406,25	2906,25	2,370	0,3102	0,0588	469,0667	0,8770
Scotland	28	3156,4	3,792	0,8812	0,5653	1968,75	1687,50	2718,75	2,321	0,1220	0,0671	584,4267	0,8902
Scotland	28	3197,4	4,085	1,0923	0,6080	1687,50	1500,00	3000,00	2,341	0,0784	0,0539	683,2800	0,8431
Scotland	28	3156,4	2,735	1,0556	0,0373	1687,50	1500,00	2250,00	1,362	0,0711	0,0558	694,4853	0,7107
Scotland	28	2828,4	3,732	0,9362	0,4640	1593,75	1406,25	2625,00	2,024	0,1257	0,0686	697,0400	0,8057
Scotland	28	3320,3	4,002	1,0372	0,5547	1781,25	1593,75	3000,00	1,719	0,0619	0,0309	1015,1467	0,8299
Scotland	28	3238,3	3,918	0,9454	0,5973	1968,75	1687,50	2812,50	1,878	0,1136	0,0568	1027,6533	0,8920
Scotland	28	3443,3	4,383	0,9930	0,6453	1875,00	1593,75	3187,50	2,497	0,0865	0,0486	1160,6880	0,8865
Scotland	28	3156,4	3,311	0,9476	0,5707	1687,50	1593,75	2437,50	1,475	0,0678	0,0452	1163,1947	0,8079
Scotland	28	3443,3	3,640	1,1423	0,6720	1781,25	1593,75	2718,75	1,679	0,0514	0,0327	1226,1173	0,7850
Scotland	28	3197,4	3,021	1,1749	0,3680	1687,50	1500,00	2437,50	1,489	0,0457	0,0274	1993,0133	0,7032
Scotland	28	3197,4	4,053	1,0464	0,5067	1781,25	1500,00	3000,00	2,088	0,0667	0,0513	2053,8187	0,8154
Scotland	28	3812,2	3,626	1,0372	0,3840	1687,50	1500,00	2812,50	1,425	0,0725	0,0570	2284,7840	0,7306
Scotland	28	3402,3	3,934	0,9821	0,5653	1687,50	1500,00	3000,00	1,470	0,0820	0,0546	2723,8560	0,7978
Scotland	28	3320,3	3,867	1,1933	0,6240	1687,50	1593,75	2812,50	1,322	0,0852	0,0628	2882,6880	0,7892
Scotland	28	3320,3	3,974	0,7894	0,3200	1593,75	1406,25	2906,25	2,302	0,1020	0,0748	3016,2080	0,7891
Scotland	29	2418,5	3,109	1,0647	0,5387	1500,00	1312,50	1968,75	1,630	0,0804	0,0503	536,4053	0,8040
Scotland	29	2459,5	3,212	1,0280	0,4373	1593,75	1406,25	2062,50	1,687	0,0885	0,0625	576,2720	0,7812
Scotland	29	2582,5	3,395	1,0647	0,5067	1593,75	1406,25	2250,00	1,782	0,0704	0,0503	582,9120	0,7739
Scotland	29	2869,4	3,521	1,1382	0,6293	1593,75	1406,25	2343,75	1,820	0,0755	0,0519	604,9760	0,8538
Scotland	29	2418,5	3,448	1,0774	0,4053	1593,75	1406,25	2250,00	1,889	0,4080	0,0498	692,1920	0,9005
Scotland	29	2705,5	3,368	1,2332	0,7040	1593,75	1406,25	2250,00	1,824	0,0693	0,0476	949,9787	0,8485
Scotland	29	2500,5	3,217	0,8957	0,5067	1593,75	1500,00	2250,00	1,838	0,0659	0,0419	956,3893	0,8204

Scotland	29	2582,5	3,470	0,9913	0,6347	1781,25	1500,00	2437,50	1,909	0,0865	0,0432	1166,8480	0,8486
Scotland	29	2705,5	3,406	1,0005	0,6080	1687,50	1500,00	2437,50	1,825	0,0802	0,0588	1177,4507	0,8235
Scotland	29	2582,5	3,494	1,1423	0,6613	1687,50	1500,00	2343,75	2,040	0,0751	0,0563	2054,9920	0,7934
Scotland	29	2828,4	3,442	1,0515	0,1067	1593,75	1500,00	2531,25	1,647	0,0612	0,0357	2081,0133	0,7959
Scotland	30	3197,4	4,343	0,8917	0,4267	1687,50	1406,25	3000,00	2,201	0,2575	0,0539	239,5787	0,9102
Scotland	30	2992,4	4,074	0,8147	0,4533	1781,25	1312,50	2718,75	2,093	0,1325	0,0530	242,2507	0,8742
Scotland	30	3279,3	4,406	0,8587	0,4853	1687,50	1312,50	3000,00	2,033	0,2062	0,0625	251,6053	0,9250
Scotland	30	3320,3	4,385	1,0128	0,5493	1875,00	1500,00	3187,50	1,838	0,2063	0,0476	264,6827	0,8942
Scotland	30	2910,4	4,046	0,8807	0,5387	1687,50	1406,25	2625,00	1,770	0,1159	0,0488	275,4667	0,8720
Scotland	30	3689,3	4,445	0,9030	0,3680	1875,00	1593,75	3375,00	2,210	0,3512	0,0357	464,1173	0,8869
Scotland	30	2951,4	3,799	0,8952	0,3893	1687,50	1500,00	2718,75	1,710	0,2695	0,0299	604,9760	0,9281
Scotland	30	4181,2	4,807	0,7926	0,4587	1875,00	1406,25	3562,50	2,405	0,2177	0,0680	833,1893	0,9116
Scotland	30	3976,2	4,535	0,8952	0,5333	1875,00	1593,75	3562,50	2,829	0,0599	0,0299	1406,5227	0,9162
Scotland	30	3730,2	4,520	1,1287	0,4587	2062,50	1500,00	3375,00	2,249	0,4265	0,0427	2074,8373	0,9431
Scotland	30	3607,3	4,505	0,9964	0,4107	1968,75	1500,00	3281,25	2,538	0,4032	0,0430	3028,3520	0,9301
Scotland	30	3478,3	4,433	1,1491	0,5493	2062,50	1593,75	3281,25	2,306	0,3364	0,0514	118,1653	0,9299
Scotland	30	3594,2	4,440	1,0342	0,4000	2062,50	1593,75	3281,25	2,221	0,3938	0,0466	238,9440	0,9016
Scotland	31	4173,9	4,563	0,8006	0,1973	2437,50	1687,50	3562,50	2,191	0,6242	0,0805	193,8080	0,9463
Scotland	31	3710,1	4,610	0,5284	0,2933	1875,00	1500,00	3375,00	1,998	0,2143	0,0408	362,6987	0,9184
Scotland	31	3768,1	4,505	0,6416	0,3307	1875,00	1593,75	3281,25	2,069	0,1849	0,0504	433,1840	0,8992
Scotland	31	3826,1	4,286	0,5014	0,2400	1875,00	1593,75	3187,50	2,078	0,1290	0,0645	467,3120	0,8602
Scotland	31	3536,2	4,285	0,8141	0,4747	1781,25	1500,00	2906,25	2,155	0,1513	0,0658	504,1920	0,8684
Scotland	31	3478,3	4,211	0,8387	0,4373	1875,00	1500,00	2906,25	2,130	0,0897	0,0449	545,3280	0,8462
Scotland	31	3420,3	4,123	0,7396	0,4053	1781,25	1500,00	2812,50	1,791	0,1241	0,0584	555,3813	0,8613
Scotland	31	3362,3	4,260	0,6634	0,3573	1687,50	1406,25	2906,25	1,954	0,1138	0,0569	576,6240	0,8699
Scotland	31	4463,8	4,651	0,9760	0,2293	2812,50	1968,75	4031,25	2,805	0,5824	0,0714	1023,4293	0,8956
Scotland	31	3652,2	4,495	0,8997	0,2560	2156,25	1593,75	3375,00	2,633	0,5000	0,0714	1029,8560	0,9167
Scotland	31	4058,0	4,744	0,9455	0,2720	2343,75	1593,75	3750,00	2,290	0,5341	0,0739	1047,1200	0,9261
Scotland	31	4463,8	4,428	0,9150	0,1760	3000,00	2250,00	4031,25	2,664	0,6550	0,4035	1060,0107	0,9181
Scotland	31	4173,9	4,721	0,5795	0,3413	2250,00	1687,50	3750,00	2,561	0,1574	0,0741	1064,2827	0,8611
Scotland	31	3884,1	4,234	0,8616	0,2080	2437,50	1687,50	3281,25	2,169	0,5875	0,1250	1070,5867	0,9313

Scotland	31	3768,1	4,507	0,8006	0,4160	1781,25	1593,75	3375,00	2,054	0,1467	0,0733	1100,8320	0,8667
Scotland	31	4637,7	4,629	0,7764	0,3947	2812,50	2156,25	4218,75	2,631	0,1586	0,0621	1178,3413	0,8000
Scotland	31	3826,1	4,230	0,9226	0,2240	2625,00	1968,75	3468,75	2,223	0,5465	0,0523	1909,7813	0,8721
Scotland	31	3768,1	4,614	0,9226	0,4160	2062,50	1500,00	3468,75	1,955	0,3895	0,0640	1919,6107	0,9186
Scotland	31	3768,1	4,297	0,8921	0,1813	2437,50	1875,00	3468,75	2,303	0,5904	0,0783	1967,2160	0,8976
Scotland	31	4173,9	4,383	0,8540	0,2187	2531,25	1875,00	3656,25	2,378	0,5786	0,0881	2041,3600	0,9371
Scotland	31	3246,4	4,135	0,8616	0,1707	2156,25	1687,50	3093,75	1,843	0,6025	0,0870	2413,7120	0,8944
Scotland	31	3768,1	4,395	0,9607	0,2187	2437,50	1781,25	3562,50	2,496	0,5698	0,0726	2425,7707	0,8939
Scotland	32	2664,5	3,619	1,1390	0,6347	1593,75	1406,25	2437,50	2,005	0,0991	0,0472	373,6747	0,8774
Scotland	32	2418,5	3,496	0,8795	0,5280	1500,00	1312,50	2156,25	1,826	0,1037	0,0488	383,3120	0,8963
Scotland	32	2377,5	3,178	1,0093	0,6507	1593,75	1312,50	2062,50	1,525	0,1064	0,0585	393,3653	0,8723
Scotland	32	2295,5	3,469	0,9948	0,6133	1500,00	1218,75	2062,50	1,536	0,1027	0,0595	397,2053	0,8595
Scotland	32	2500,5	3,266	0,8795	0,3787	1593,75	1406,25	2156,25	1,455	0,2622	0,0610	410,8853	0,8537
Scotland	32	2541,5	3,445	0,9660	0,4000	1500,00	1218,75	2062,50	1,826	0,0722	0,0444	464,3200	0,7778
Scotland	32	3074,4	3,909	1,0909	0,4000	1593,75	1312,50	2625,00	1,665	0,0784	0,0490	610,9227	0,7941
Scotland	32	2541,5	3,265	0,9277	0,5920	1781,25	1500,00	2343,75	1,584	0,0867	0,0520	766,2827	0,8786
Scotland	32	2828,4	3,513	0,9804	0,6347	1593,75	1406,25	2343,75	1,879	0,0984	0,0492	798,4053	0,8743
Scotland	32	2664,5	3,787	1,0603	0,4907	1687,50	1406,25	2531,25	1,945	0,3434	0,0808	928,8533	0,9192
Scotland	32	2828,4	3,823	1,0093	0,5173	1781,25	1593,75	2812,50	2,317	0,1217	0,0794	931,0347	0,8624
Scotland	32	2623,5	3,392	1,2234	0,4267	1500,00	1312,50	2156,25	1,616	0,3755	0,0961	973,8773	0,8341
Scotland	32	2459,5	3,583	1,0501	0,3467	1687,50	1406,25	2343,75	1,961	0,4490	0,0561	1002,8320	0,9031
Scotland	32	3033,4	4,043	1,0603	0,4160	1687,50	1406,25	2812,50	2,237	0,1010	0,0606	1102,7360	0,8384
Scotland	32	2500,5	3,348	1,0093	0,5280	1687,50	1406,25	2250,00	1,907	0,2234	0,0532	1200,1653	0,8883
Scotland	32	2336,5	3,298	1,0093	0,5493	1593,75	1312,50	2156,25	1,600	0,1905	0,0529	1215,7760	0,9048
Scotland	32	2295,5	3,256	1,0093	0,4960	1687,50	1406,25	2156,25	1,621	0,2275	0,0635	1229,7387	0,8836
Scotland	32	2418,5	3,275	0,9175	0,4213	1687,50	1406,25	2250,00	1,625	0,2865	0,0468	1256,4107	0,9006
Scotland	32	2541,5	3,597	0,9379	0,4320	1687,50	1406,25	2437,50	1,640	0,3391	0,0460	1264,8747	0,9310
Scotland	32	2541,5	3,373	0,9175	0,5173	1687,50	1500,00	2343,75	1,622	0,0936	0,0468	1277,7333	0,8480
Scotland	32	2582,5	3,500	0,7646	0,5280	1593,75	1406,25	2250,00	1,891	0,0845	0,0493	1345,0293	0,9366
Scotland	32	2582,5	3,413	0,9583	0,5493	1687,50	1500,00	2250,00	1,929	0,1292	0,0393	1351,3387	0,8539
Scotland	33	2418,5	2,982	0,7935	0,4640	1500,00	1312,50	1968,75	1,608	0,0676	0,0338	3,4347	0,8514

Scotland	33	2500,5	3,257	0,9970	0,6080	1500,00	1312,50	2062,50	1,589	0,1022	0,0591	22,8000	0,8925
Scotland	33	2541,5	3,030	1,0513	0,6027	1593,75	1406,25	2062,50	1,535	0,0918	0,0510	72,8853	0,8316
Scotland	33	2664,5	2,947	1,0920	0,6347	1500,00	1406,25	1968,75	1,528	0,0887	0,0542	113,4720	0,9064
Scotland	33	2541,5	2,714	1,0241	0,5920	1500,00	1312,50	1875,00	1,501	0,0838	0,0471	118,7787	0,8482
Scotland	33	2664,5	2,945	1,1259	0,6347	1500,00	1406,25	1968,75	1,422	0,1000	0,0619	146,4320	0,8810
Scotland	33	2541,5	3,069	1,0648	0,4000	1500,00	1312,50	1968,75	1,555	0,3467	0,0452	165,6213	0,8794
Scotland	33	2459,5	2,768	0,9495	0,5227	1500,00	1312,50	1875,00	1,492	0,1017	0,0565	169,8453	0,8136
Scotland	33	2541,5	2,818	0,9428	0,5173	1500,00	1406,25	1875,00	1,494	0,0914	0,0514	181,0880	0,8171
Scotland	33	2541,5	2,832	0,8139	0,4320	1500,00	1312,50	1875,00	1,490	0,0724	0,0526	187,8667	0,7368
Scotland	33	2582,5	2,833	1,0513	0,6560	1500,00	1312,50	1875,00	1,396	0,1071	0,0510	220,9547	0,8878
Scotland	33	2295,5	3,016	0,8817	0,4373	1500,00	1312,50	1968,75	1,520	0,0793	0,0488	243,4240	0,7622
Scotland	33	2377,5	3,180	0,8681	0,5120	1500,00	1218,75	1968,75	1,336	0,0988	0,0370	246,8160	0,8395
Scotland	33	2418,5	3,072	0,9495	0,5600	1500,00	1031,25	1875,00	1,570	0,0621	0,0339	252,2613	0,8136
Scotland	33	2459,5	2,965	0,9563	0,5600	1500,00	1312,50	1968,75	1,497	0,0838	0,0391	257,9573	0,8380
Scotland	33	2418,5	2,932	0,9495	0,4160	1500,00	1312,50	1968,75	1,402	0,1582	0,0339	264,8320	0,7797
Scotland	33	2418,5	2,937	0,9224	0,4693	1500,00	1312,50	1968,75	1,555	0,0930	0,0407	273,0027	0,7326
Scotland	33	2623,5	2,935	0,9021	0,4587	1500,00	1312,50	1875,00	1,405	0,1131	0,0357	280,0800	0,7440
Scotland	33	2459,5	3,283	0,8953	0,5547	1500,00	1031,25	1968,75	1,560	0,0958	0,0479	304,5547	0,8922
Scotland	33	2500,5	3,280	0,9021	0,4000	1500,00	1218,75	1968,75	1,344	0,0893	0,0595	309,1253	0,8036
Scotland	33	2295,5	2,922	0,8614	0,4800	1500,00	1218,75	1875,00	1,548	0,1000	0,0563	312,4053	0,7875
Scotland	33	2500,5	3,283	1,0513	0,4373	1500,00	1031,25	2062,50	0,952	0,3112	0,0510	336,5707	0,8724
Scotland	33	2459,5	3,204	0,9021	0,5173	1500,00	1218,75	1968,75	1,138	0,0893	0,0417	341,3653	0,8274
Scotland	33	2541,5	3,042	1,0648	0,3733	1593,75	1406,25	1968,75	1,425	0,4121	0,0553	380,7093	0,9095
Scotland	33	2418,5	2,956	1,0038	0,5973	1500,00	1312,50	1875,00	1,553	0,0909	0,0535	384,7787	0,8717
Scotland	33	2459,5	3,023	0,8681	0,4693	1500,00	1312,50	1968,75	1,516	0,0741	0,0370	390,4533	0,7901
Scotland	34	3095,0	3,460	0,5835	0,3360	1500,00	1312,50	2343,75	1,489	0,1204	0,0648	5,7760	0,8981
Scotland	34	3475,1	4,220	0,8603	0,5227	1687,50	1406,25	2812,50	2,101	0,1180	0,0435	127,2800	0,8758
Scotland	34	3583,7	4,132	0,8362	0,4853	1687,50	1218,75	2625,00	2,092	0,1667	0,0385	139,7440	0,9231
Scotland	34	3095,0	4,291	0,9024	0,3360	1593,75	1312,50	2812,50	1,824	0,4048	0,0655	179,1680	0,9226
Scotland	34	2986,4	3,988	0,9204	0,4800	1687,50	1406,25	2625,00	1,680	0,2209	0,0523	204,6080	0,9070
Scotland	34	3312,2	4,150	0,9024	0,5440	1593,75	1218,75	2812,50	1,681	0,1310	0,0476	208,2933	0,8929

Scotland	34	3583,7	4,155	0,8482	0,2347	1875,00	1500,00	2906,25	2,345	0,4340	0,0377	234,3947	0,8050
Scotland	34	3475,1	4,239	0,8663	0,5440	1687,50	1406,25	2812,50	2,134	0,0745	0,0186	236,6880	0,8634
Scotland	34	3529,4	4,170	0,8603	0,6027	1593,75	1406,25	2906,25	1,561	0,1125	0,0437	453,4187	0,9187
Scotland	34	3855,2	4,313	0,8181	0,4373	1781,25	1500,00	3000,00	1,816	0,1699	0,0523	643,5307	0,8693
Scotland	34	3583,7	4,200	1,1310	0,5013	1687,50	1406,25	2812,50	2,092	0,3081	0,0284	645,0880	0,9052
Scotland	34	3909,5	3,731	0,7219	0,2880	1687,50	1593,75	2812,50	1,531	0,2000	0,0667	649,9147	0,7852
Scotland	34	3529,4	3,849	0,6557	0,3893	1781,25	1500,00	2718,75	1,666	0,0902	0,0410	656,7253	0,8443
Scotland	34	3257,9	3,389	0,6377	0,3253	1781,25	1500,00	2343,75	1,626	0,1186	0,0593	661,4400	0,7966
Scotland	34	3746,6	4,355	0,8001	0,5013	1781,25	1500,00	3093,75	1,864	0,1074	0,0470	684,9173	0,8792
Scotland	35	2582,5	3,348	0,8750	0,5760	1593,75	1406,25	2250,00	1,398	0,1043	0,0368	13,9787	0,9264
Scotland	35	2172,6	2,615	0,8883	0,5280	1500,00	1406,25	1875,00	1,411	0,0909	0,0485	24,4907	0,8364
Scotland	35	2295,5	2,861	0,9612	0,5867	1500,00	1406,25	1875,00	1,346	0,1167	0,0333	55,6000	0,9111
Scotland	35	2336,5	2,676	0,8949	0,5707	1500,00	1406,25	1875,00	1,323	0,1325	0,0663	60,3200	0,9157
Scotland	35	2336,5	2,813	0,9811	0,2933	1500,00	1406,25	1968,75	1,324	0,4754	0,0492	91,7120	0,9235
Scotland	35	2459,5	3,124	0,9480	0,6293	1593,75	1406,25	2062,50	1,460	0,1534	0,0568	96,4907	0,9375
Scotland	35	2377,5	2,554	0,9480	0,5280	1500,00	1406,25	1875,00	1,277	0,0904	0,0565	123,8560	0,8588
Scotland	35	2418,5	2,943	0,8220	0,5120	1500,00	1406,25	1968,75	1,437	0,1176	0,0588	133,2427	0,9020
Scotland	35	2254,5	2,607	0,8883	0,5333	1500,00	1312,50	1875,00	1,275	0,1205	0,0663	158,1600	0,8614
Scotland	35	2459,5	2,855	0,8419	0,4373	1500,00	1406,25	2062,50	1,381	0,0955	0,0510	164,1227	0,8790
Scotland	35	2377,5	2,902	0,9678	0,6453	1593,75	1500,00	2062,50	1,287	0,0994	0,0552	182,5120	0,8950
Scotland	35	2541,5	2,953	0,9082	0,5867	1593,75	1500,00	2062,50	1,491	0,1353	0,0706	188,4853	0,9000
Scotland	35	2459,5	3,048	0,8750	0,6080	1593,75	1500,00	2062,50	1,345	0,1402	0,0610	209,4933	0,9146
Scotland	35	2500,5	3,045	1,0408	0,3093	1593,75	1500,00	2062,50	1,558	0,5155	0,0670	251,7387	0,9072
Scotland	35	2459,5	3,045	0,9546	0,6720	1593,75	1406,25	2062,50	1,536	0,0843	0,0393	256,9120	0,9270
Scotland	35	2213,6	2,399	0,9148	0,5173	1500,00	1406,25	1781,25	1,282	0,0882	0,0471	270,1333	0,8706
Scotland	35	2254,5	2,539	0,9082	0,5067	1500,00	1406,25	1875,00	1,318	0,1118	0,0647	277,9893	0,8412
Scotland	35	2254,5	2,698	1,0474	0,2613	1500,00	1406,25	1875,00	1,356	0,4923	0,1128	298,8107	0,8821
Scotland	35	2254,5	2,608	0,9281	0,5493	1500,00	1406,25	1875,00	1,317	0,1098	0,0520	307,8880	0,8671
Scotland	35	2377,5	2,907	0,8618	0,4373	1500,00	1406,25	1968,75	1,454	0,1615	0,0621	313,4347	0,8323
Scotland	35	2500,5	2,990	0,9015	0,5227	1593,75	1406,25	2062,50	1,590	0,1369	0,0655	323,0667	0,8750
Scotland	35	2418,5	2,623	0,9281	0,5493	1500,00	1312,50	1781,25	1,363	0,0925	0,0520	361,8187	0,8728

Scotland	35	2418,5	2,495	0,9082	0,5067	1500,00	1406,25	1781,25	1,312	0,1006	0,0414	381,6480	0,8580
Scotland	36	2929,3	3,620	0,9605	0,5547	1687,50	1312,50	2343,75	1,963	0,0726	0,0447	609,4880	0,8212
Scotland	36	2621,0	3,677	1,0122	0,4907	1406,25	1125,00	2156,25	1,729	0,0745	0,0532	613,0080	0,7766
Scotland	36	2621,0	3,205	0,7953	0,0587	1593,75	1312,50	2062,50	1,903	0,0946	0,0743	744,8533	0,7635
Scotland	36	2723,8	3,569	1,1981	0,6080	1500,00	1218,75	2250,00	1,756	0,2511	0,0538	787,5413	0,9013
Scotland	36	2723,8	3,508	1,1774	0,7040	1593,75	1406,25	2250,00	1,735	0,1136	0,0773	828,9973	0,8364
Scotland	36	2826,6	3,492	1,2600	0,3040	1687,50	1312,50	2343,75	1,680	0,4788	0,0424	858,9600	0,8602
Scotland	36	2518,2	3,234	1,0225	0,4693	1406,25	1218,75	2062,50	1,634	0,0681	0,0471	902,6827	0,7853
Scotland	36	2518,2	3,402	0,9812	0,4533	1406,25	1218,75	2062,50	1,415	0,0929	0,0710	909,3547	0,7760
Scotland	36	2621,0	3,218	1,0535	0,4533	1406,25	1218,75	2062,50	1,396	0,0612	0,0408	939,2587	0,7347
Scotland	36	2672,4	3,439	1,2187	0,3840	1593,75	1218,75	2156,25	1,666	0,3877	0,0529	1002,9600	0,8458
Scotland	36	2672,4	3,644	1,2084	0,3840	1593,75	1218,75	2250,00	1,697	0,4779	0,0619	1029,3813	0,9292
Scotland	36	2826,6	3,628	1,2807	0,3840	1500,00	1218,75	2250,00	1,590	0,3808	0,0460	1191,6747	0,8828
Scotland	36	2723,8	3,676	1,1568	0,3360	1500,00	1218,75	2250,00	1,785	0,4120	0,0417	1199,0773	0,8194
Scotland	37	2415,4	3,390	0,9640	0,6507	1593,75	1312,50	2156,25	1,693	0,0611	0,0389	312,4480	0,8722
Scotland	37	2775,2	3,664	0,9932	0,4747	1500,00	1218,75	2343,75	1,643	0,0649	0,0486	408,0213	0,8270
Scotland	37	2518,2	3,393	1,3220	0,4267	1593,75	1218,75	2156,25	1,697	0,4049	0,0445	495,7493	0,8462
Scotland	37	2672,4	3,298	1,1981	0,3307	1593,75	1312,50	2156,25	1,676	0,3661	0,0536	514,1067	0,8170
Scotland	37	2723,8	3,822	1,3943	0,4373	1593,75	1312,50	2437,50	1,807	0,3602	0,0498	536,8587	0,8966
Scotland	37	2621,0	3,504	1,3323	0,3840	1593,75	1218,75	2156,25	1,072	0,4819	0,0482	692,0213	0,8956
Scotland	37	2723,8	3,352	1,3323	0,1760	1593,75	1406,25	2250,00	1,794	0,5341	0,0442	944,9173	0,7711
Russia	38	2233,6	3,170	1,0483	0,5600	1593,75	1312,50	1968,75	1,598	0,1122	0,0408	45,5093	0,8418
Russia	38	2146,0	2,747	0,9044	0,4747	1312,50	1218,75	1687,50	1,314	0,1006	0,0651	49,5680	0,8817
Russia	38	2365,0	3,300	0,9147	0,3733	1500,00	1406,25	2250,00	1,375	0,2105	0,0819	73,1413	0,8772
Russia	38	2102,2	2,989	1,0380	0,5760	1406,25	1218,75	1875,00	1,502	0,0722	0,0515	111,6640	0,7784
Russia	38	2321,2	3,230	0,8427	0,3147	1500,00	1312,50	2062,50	1,630	0,2548	0,0701	141,3547	0,8599
Russia	38	2189,8	3,170	1,1408	0,5173	1500,00	1218,75	1968,75	1,590	0,0986	0,0657	159,6853	0,7371
Russia	38	2277,4	3,335	1,0791	0,4853	1500,00	1218,75	2062,50	1,608	0,1144	0,0498	166,7093	0,7960
Russia	39	2114,3	3,094	1,0668	0,2773	1312,50	656,25	1781,25	0,360	0,4070	0,0804	363,1467	0,8191
Russia	39	2342,9	3,246	1,0324	0,2773	1312,50	1125,00	2062,50	1,377	0,3802	0,0781	392,1173	0,8333
Russia	39	2400,0	3,334	1,1194	0,4960	562,50	562,50	1875,00	0,631	0,3062	0,1005	462,1013	0,8517

Russia	39	2342,9	3,116	1,0220	0,5973	1312,50	1218,75	1968,75	1,341	0,1526	0,1105	491,8560	0,8526
Russia	39	1942,9	2,296	1,1437	0,5547	1312,50	1218,75	1593,75	1,312	0,1729	0,0888	660,6827	0,8224
Russia	40	3160,0	4,220	1,0277	0,4053	1500,00	1312,50	2812,50	2,120	0,3490	0,0781	1870,0640	0,8646
Russia	40	2808,9	4,177	0,8894	0,3467	1500,00	1125,00	2625,00	1,873	0,3576	0,0788	1874,1227	0,8545
Russia	40	2691,9	4,262	0,8894	0,3787	1406,25	750,00	2531,25	1,223	0,3434	0,1084	1892,6240	0,9157
Russia	40	2808,9	4,110	1,1661	0,3040	1500,00	1031,25	2531,25	1,489	0,3899	0,1009	1898,5120	0,7982
Russia	40	2574,8	3,994	1,1661	0,3040	1593,75	843,75	2437,50	1,339	0,4700	0,1198	1903,7547	0,8387
Russia	40	2691,9	4,026	0,8894	0,3093	1593,75	1218,75	2531,25	1,587	0,4096	0,0783	1907,1733	0,8554
Russia	40	2808,9	4,032	0,9091	0,4053	1500,00	1031,25	2437,50	1,357	0,2882	0,1000	1913,4507	0,8294
Russia	40	2808,9	4,095	1,0475	0,3413	1593,75	1312,50	2531,25	2,085	0,4513	0,0872	1925,9040	0,8718
Russia	40	2926,0	4,139	1,0672	0,3147	1500,00	1218,75	2625,00	2,075	0,4422	0,1206	1933,0773	0,8392
Russia	40	2691,9	3,848	1,0870	0,3360	1500,00	1312,50	2437,50	1,207	0,4631	0,1084	2149,5360	0,8867
Russia	40	2926,0	3,891	1,0870	0,2880	1593,75	1312,50	2437,50	1,829	0,4010	0,0941	2161,6693	0,7871
Russia	40	3043,0	3,868	1,2056	0,3520	1500,00	1312,50	2531,25	1,790	0,3956	0,0844	2181,9787	0,8044
Russia	40	2691,9	3,859	1,1265	0,2240	1593,75	1312,50	2437,50	1,550	0,4857	0,0905	2190,9173	0,8000
Russia	41	2647,1	3,053	1,0062	0,4267	1218,75	1125,00	1875,00	1,388	0,1117	0,0691	562,3787	0,7606
Russia	41	2647,1	3,127	1,0342	0,4267	1312,50	1218,75	1968,75	1,334	0,1823	0,1250	574,0480	0,7812
Russia	41	2941,2	2,683	1,0621	0,3200	1312,50	1218,75	1968,75	1,289	0,1818	0,1414	656,3680	0,7121
Russia	41	3117,6	3,010	1,0621	0,3573	1312,50	1218,75	2250,00	1,394	0,1717	0,1212	667,5040	0,7525
Russia	41	3352,9	3,943	1,0342	0,4693	1312,50	1125,00	2531,25	1,327	0,2124	0,1036	689,2587	0,8290
Russia	41	3294,1	3,927	0,9783	0,4907	1218,75	1031,25	2531,25	1,684	0,1374	0,0659	697,8773	0,8022
Russia	41	3136,9	2,984	1,0475	0,3147	1312,50	1125,00	1968,75	1,469	0,2154	0,1282	1087,8667	0,7333
Russia	41	3074,1	2,607	0,8894	0,2027	1312,50	1218,75	1781,25	1,286	0,1394	0,1030	1156,2293	0,7030
Russia	42	2258,6	3,003	1,0870	0,5333	1218,75	1125,00	1781,25	1,471	0,1330	0,0690	1451,3333	0,8276
Russia	42	2258,6	2,578	1,0080	0,4587	1312,50	1218,75	1781,25	1,323	0,1436	0,1011	1468,2347	0,8085
Russia	42	2133,1	2,998	1,0080	0,5440	1312,50	1125,00	1781,25	1,394	0,1436	0,0851	1473,6853	0,8245
Russia	42	2384,0	3,116	1,2056	0,4213	1218,75	937,50	1875,00	1,411	0,1244	0,0711	1491,7067	0,7689
Russia	42	2384,0	3,249	1,1858	0,5440	1218,75	937,50	1875,00	1,218	0,1584	0,0724	1517,3547	0,8145
Russia	42	2572,2	2,752	1,2847	0,5067	1312,50	1218,75	1875,00	1,298	0,1000	0,0542	1611,6853	0,7500
Russia	43	2258,8	2,706	1,3509	0,2027	1500,00	1312,50	1875,00	1,082	0,5119	0,3968	41,3120	0,7897
Russia	43	2070,6	2,661	1,0947	0,3893	1406,25	1218,75	1687,50	1,356	0,3137	0,0882	45,1413	0,8137

Russia	43	2070,6	2,349	1,3043	0,1760	1406,25	1312,50	1687,50	1,334	0,4650	0,1317	85,2213	0,6996
Russia	43	2164,7	2,282	1,2112	0,1760	1500,00	1406,25	1687,50	1,298	0,5133	0,3496	140,3893	0,8186
Russia	43	2117,6	2,604	1,1646	0,2133	1500,00	1406,25	1875,00	1,570	0,5550	0,4083	174,3093	0,8440
Russia	43	2211,8	2,263	1,2112	0,1600	1593,75	1500,00	1781,25	1,275	0,5929	0,5000	202,1600	0,8274
Russia	43	2258,8	2,360	1,1646	0,1760	1500,00	1406,25	1781,25	1,268	0,5346	0,4654	228,5547	0,7880
Russia	43	2305,9	2,597	1,3276	0,2347	1593,75	1500,00	1968,75	1,475	0,5685	0,4798	278,7947	0,8750
Russia	43	2211,8	2,722	1,2578	0,1280	1593,75	1406,25	1875,00	1,338	0,6043	0,4426	322,8320	0,8213
Russia	43	2117,6	2,315	1,2345	0,1760	1593,75	1500,00	1781,25	1,362	0,4957	0,3957	383,3067	0,7391
Russia	43	2023,5	2,372	1,1646	0,1493	1500,00	1406,25	1781,25	1,290	0,5000	0,0780	450,7467	0,7752
Russia	43	1882,4	3,065	1,2345	0,2933	1406,25	1218,75	1875,00	1,548	0,4545	0,0996	477,2320	0,8485
Russia	43	1788,2	2,477	1,1413	0,1973	1406,25	1218,75	1687,50	1,312	0,4366	0,1033	495,8613	0,7512
Russia	43	1929,4	3,083	1,0481	0,4267	1312,50	1125,00	1875,00	0,793	0,1224	0,0765	498,6400	0,7806
Russia	43	1788,2	2,660	0,9550	0,2347	1406,25	1218,75	1781,25	0,927	0,3989	0,1067	526,2987	0,8315
Russia	43	1835,3	2,669	1,1879	0,3253	1406,25	1125,00	1781,25	0,783	0,4820	0,1081	556,2720	0,8198
Russia	43	2164,7	3,070	1,0016	0,4960	1406,25	1218,75	1875,00	1,492	0,2032	0,1337	559,6480	0,8289
Russia	43	2258,8	2,828	1,1413	0,1760	1593,75	1406,25	1968,75	1,416	0,6009	0,4554	968,3253	0,8451
Russia	43	2352,9	3,102	1,1879	0,2080	1406,25	1218,75	1875,00	1,394	0,5045	0,1126	971,4453	0,8514
Russia	43	2117,6	3,125	1,2578	0,2187	1500,00	1218,75	1875,00	1,383	0,4851	0,1149	1085,0453	0,7957
Russia	44	1976,5	2,339	1,2188	0,5653	1312,50	1218,75	1593,75	1,292	0,1410	0,1013	34,9387	0,7665
Russia	44	2070,6	2,432	1,1364	0,5653	1406,25	1218,75	1687,50	1,295	0,0896	0,0566	81,5520	0,7925
Russia	44	1741,2	2,586	1,2023	0,5493	1312,50	1125,00	1687,50	1,312	0,1518	0,0938	105,7387	0,7679
Russia	44	1976,5	2,356	0,8729	0,4000	1406,25	1218,75	1687,50	1,264	0,1043	0,0736	108,7680	0,8098
Russia	44	1976,5	2,553	1,0541	0,4747	1312,50	1218,75	1593,75	1,363	0,1320	0,0761	113,0240	0,8020
Russia	44	1929,4	2,467	1,0870	0,5120	1406,25	1218,75	1687,50	1,140	0,0739	0,0493	247,1573	0,7833
Russia	44	2070,6	2,746	1,2188	0,5600	1312,50	1125,00	1687,50	1,403	0,0925	0,0573	259,8507	0,8018
Russia	44	2070,6	2,457	1,0541	0,4747	1312,50	1218,75	1593,75	1,310	0,0964	0,0609	290,5707	0,7513
Russia	44	1788,2	2,375	0,8894	0,4587	1312,50	1218,75	1593,75	1,384	0,0904	0,0542	294,7893	0,8193
Russia	44	1976,5	2,885	1,0705	0,4480	1218,75	1031,25	1687,50	1,447	0,1300	0,0750	315,3440	0,8150
Russia	44	2117,6	2,343	1,1529	0,4907	1312,50	1218,75	1593,75	1,284	0,0744	0,0558	336,3520	0,7349
Russia	44	1929,4	2,935	1,0541	0,5120	1312,50	843,75	1593,75	1,329	0,1122	0,0612	462,9280	0,8520
Russia	44	1835,3	2,686	1,0705	0,5067	1312,50	1125,00	1593,75	1,352	0,1400	0,0700	547,1840	0,8250

Russia	44	1819,4	2,162	0,9388	0,3733	1312,50	1218,75	1500,00	1,280	0,1029	0,0686	649,8720	0,7714
Russia	44	2007,6	2,599	0,9717	0,1547	1312,50	1125,00	1593,75	1,364	0,1050	0,0663	653,2480	0,7459
Russia	44	1944,9	2,447	1,0541	0,4693	1312,50	1218,75	1593,75	1,354	0,1429	0,0867	670,7467	0,7857
Russia	44	1944,9	2,456	0,9717	0,4320	1312,50	1218,75	1687,50	1,341	0,1326	0,0884	684,0320	0,8287
Russia	44	2007,6	2,263	1,0705	0,5333	1312,50	1218,75	1593,75	1,287	0,1300	0,0600	763,1147	0,8000
Russia	45	1757,1	2,695	0,9243	0,4960	1218,75	1031,25	1500,00	1,290	0,1163	0,0640	10,0800	0,8198
Russia	45	1799,9	2,946	1,0188	0,5920	1312,50	937,50	1593,75	1,561	0,1105	0,0526	69,9893	0,8684
Russia	45	1928,5	3,319	0,9663	0,4373	1125,00	843,75	1687,50	1,376	0,2500	0,0778	89,2160	0,9000
Russia	45	1971,3	3,090	0,8928	0,1600	1218,75	1031,25	1687,50	1,282	0,4759	0,0964	100,0693	0,8735
Russia	45	1757,1	2,405	0,9558	0,1280	1312,50	1125,00	1593,75	1,321	0,5225	0,0674	160,1920	0,8315
Russia	46	2206,6	2,806	0,8545	0,1547	1406,25	1218,75	1781,25	1,347	0,5346	0,1069	102,0800	0,8553
Russia	46	2166,5	3,067	1,0277	0,2773	1406,25	1125,00	1781,25	1,352	0,4921	0,1099	129,3173	0,8848
Russia	46	2286,8	2,932	1,1085	0,2240	1406,25	1218,75	1875,00	1,531	0,5169	0,1498	285,7387	0,8406
Russia	46	2126,3	3,014	1,0046	0,5760	1312,50	1125,00	1781,25	1,407	0,1283	0,0749	291,0080	0,8289
Russia	46	2086,2	2,911	0,9930	0,2240	1406,25	1218,75	1781,25	1,236	0,4865	0,1027	300,2187	0,8216
Russia	46	2400,0	2,928	1,1474	0,1973	1406,25	1312,50	1875,00	1,335	0,4673	0,0748	37,1200	0,7757
Russia	47	2362,4	3,813	1,1772	0,2613	1500,00	1031,25	2250,00	1,756	0,4864	0,0727	61,2213	0,8500
Russia	47	2362,4	3,878	1,0285	0,2987	1406,25	937,50	2250,00	1,934	0,3698	0,0521	77,5413	0,8385
Russia	47	2577,2	3,618	1,2887	0,2773	1406,25	937,50	2343,75	1,650	0,4250	0,1125	140,2240	0,8250
Russia	47	2362,4	3,819	1,2019	0,6293	1218,75	1031,25	2156,25	1,673	0,1339	0,0670	142,8053	0,8884
Russia	47	2201,3	3,306	0,8674	0,3840	1312,50	1125,00	1968,75	1,536	0,2160	0,1111	145,9627	0,8395
Russia	47	2308,7	3,645	1,1028	0,6133	1218,75	1031,25	2062,50	1,402	0,1456	0,0728	191,0027	0,8398
Russia	48	2335,1	2,958	0,4743	0,2187	1406,25	1312,50	1875,00	1,787	0,1818	0,1136	16,1920	0,7386
Russia	48	2270,3	3,185	0,5617	0,2720	1406,25	1218,75	1968,75	1,417	0,1635	0,0769	17,5680	0,7404
Russia	48	2400,0	3,085	0,7614	0,3520	1406,25	1312,50	1968,75	1,539	0,2113	0,0563	34,6347	0,7817
Russia	48	2659,5	3,313	0,6490	0,2667	1406,25	1312,50	2062,50	1,551	0,1333	0,0833	38,4747	0,7000
Russia	48	2464,9	3,395	0,8238	0,2613	1500,00	1312,50	2062,50	1,741	0,4314	0,0588	59,2213	0,8562
Russia	49	2534,5	3,288	1,0984	0,5227	1500,00	1312,50	2062,50	1,852	0,1122	0,0732	23,1573	0,8585
Russia	49	2379,3	3,163	0,8238	0,2720	1500,00	1312,50	1968,75	1,789	0,1494	0,1039	25,8080	0,7857
Russia	49	2534,5	3,578	0,8113	0,3893	1593,75	1312,50	2250,00	2,069	0,1457	0,0993	64,0640	0,8278
Russia	49	2379,3	3,599	0,8363	0,5973	1312,50	1125,00	2062,50	1,642	0,1154	0,0449	81,1520	0,9679

Russia	49	2534,5	3,520	0,8113	0,3840	1500,00	1312,50	2156,25	1,737	0,1523	0,0993	82,6933	0,7947
Russia	49	2556,5	3,624	0,9817	0,5120	1687,50	1312,50	2343,75	2,057	0,1967	0,0546	77,6320	0,8907
Russia	49	2347,8	3,537	0,9239	0,5013	1406,25	1218,75	2062,50	2,069	0,0465	0,0116	80,4907	0,8023
Russia	49	2452,2	3,625	1,0279	0,7093	1500,00	1218,75	2250,00	1,475	0,0990	0,0625	121,7547	0,9062
Russia	49	2243,5	3,311	0,9239	0,5120	1593,75	1312,50	2156,25	1,693	0,0930	0,0640	191,9627	0,8663
Russia	49	2400,0	3,402	0,9239	0,5600	1593,75	1218,75	2156,25	2,142	0,0930	0,0581	202,7947	0,8895
Russia	49	2347,8	3,414	1,2126	0,7147	1500,00	1312,50	2156,25	1,800	0,0885	0,0575	235,4080	0,8584
Russia	49	2400,0	3,347	1,2011	0,6773	1406,25	1218,75	2062,50	1,828	0,0848	0,0446	279,9520	0,8214
Russia	50	2552,1	3,892	1,0859	0,7093	1500,00	1218,75	2437,50	1,931	0,1139	0,0594	161,6960	0,9158
Russia	50	2503,1	3,626	0,9731	0,6027	1406,25	1218,75	2250,00	1,725	0,1050	0,0773	180,9013	0,9006
Russia	50	2748,5	3,712	0,6769	0,2613	1593,75	1406,25	2437,50	1,961	0,1920	0,1120	192,9333	0,8240
Russia	50	2208,6	3,207	0,9308	0,3413	1500,00	1312,50	2062,50	1,345	0,0977	0,0690	229,2053	0,7759
Russia	50	2503,1	3,541	1,1705	0,2773	1687,50	1500,00	2343,75	1,883	0,4932	0,0913	231,8133	0,8813
Russia	50	2257,7	3,544	0,9167	0,5333	1406,25	1125,00	2062,50	1,732	0,1228	0,0702	279,3653	0,8538
Russia	51	2251,5	3,649	1,2128	0,4480	1312,50	1031,25	2062,50	1,644	0,3717	0,0929	39,3227	0,8938
Russia	51	2538,9	3,620	1,3139	0,6613	1312,50	1031,25	2062,50	1,730	0,1306	0,0776	42,8213	0,8327
Russia	51	2586,8	3,772	1,2128	0,5013	1218,75	937,50	2062,50	1,849	0,1549	0,0841	46,1973	0,7920
Russia	51	2586,8	3,797	1,5160	0,6773	1312,50	1031,25	2156,25	1,664	0,1444	0,0704	115,7173	0,7923
Russia	51	2634,7	3,670	1,1623	0,5440	1218,75	1031,25	2062,50	1,702	0,1751	0,1060	119,5253	0,8525
Russia	51	2730,5	3,804	1,1370	0,5120	1218,75	1031,25	2250,00	1,686	0,2113	0,1268	124,3520	0,9390
Russia	51	2155,7	3,423	0,8338	0,3360	1312,50	1031,25	1875,00	1,764	0,1355	0,0581	137,5200	0,8258
Russia	52	2202,9	2,631	1,0766	0,5227	1406,25	1218,75	1687,50	1,364	0,1244	0,0697	98,4533	0,7711
Russia	52	2087,0	2,502	1,0527	0,4640	1406,25	1218,75	1687,50	1,324	0,1320	0,1015	105,5147	0,7462
Russia	52	2087,0	2,478	0,9570	0,1173	1312,50	1218,75	1687,50	1,388	0,1173	0,0894	113,4720	0,6872
Russia	52	2087,0	2,760	1,0527	0,5387	1406,25	1218,75	1687,50	1,361	0,0964	0,0761	130,2560	0,7665
Russia	52	2202,9	2,532	0,9809	0,5227	1406,25	1218,75	1687,50	1,345	0,1202	0,0874	142,8960	0,7596
Russia	52	2144,9	2,853	0,9570	0,4693	1312,50	1218,75	1781,25	1,361	0,1732	0,1285	147,7493	0,8492
Russia	52	2087,0	2,952	0,9570	0,4640	1312,50	1218,75	1781,25	1,084	0,1676	0,1061	151,2373	0,7933
Russia	53	2425,5	3,621	1,2907	0,4373	1593,75	1312,50	2343,75	1,792	0,4149	0,1245	861,0933	0,8423
Russia	53	2170,2	3,417	1,2558	0,4107	1500,00	1218,75	2156,25	1,687	0,3830	0,1234	871,7973	0,8596
Russia	53	2744,7	4,043	1,1163	0,3787	1687,50	1312,50	2625,00	2,287	0,4258	0,1100	881,1413	0,8373

Russia	53	2617,0	3,897	1,2558	0,3733	1687,50	1312,50	2531,25	1,984	0,4658	0,1368	917,0133	0,8376
Russia	53	2553,2	3,840	1,2209	0,6080	1687,50	1312,50	2531,25	1,407	0,2719	0,0746	943,4560	0,8421
Russia	53	2553,2	3,798	0,9767	0,2933	1781,25	1406,25	2531,25	1,632	0,5137	0,1421	958,5867	0,8907
Russia	53	2234,0	3,276	0,8140	0,3147	1593,75	1406,25	2156,25	1,312	0,4079	0,1316	1024,2880	0,8750
Russia	53	2106,4	2,935	0,9373	0,4160	1406,25	1218,75	1875,00	1,348	0,1200	0,0914	1054,2187	0,7200
Russia	53	2425,5	3,528	1,0360	0,3093	1687,50	1406,25	2343,75	2,120	0,4611	0,1399	1078,2080	0,8342
Russia	53	2680,9	3,767	1,1840	0,3253	1687,50	1406,25	2531,25	1,884	0,5339	0,1493	1104,7413	0,9050
Russia	53	2234,0	3,033	1,2087	0,2560	1500,00	1312,50	2062,50	1,668	0,4356	0,0889	1190,7573	0,7689
Russia	53	2425,5	2,989	1,0607	0,2667	1406,25	1218,75	1968,75	1,317	0,4264	0,0863	1205,1947	0,7970
Russia	53	2042,6	2,721	1,1100	0,5227	1406,25	1218,75	1781,25	1,318	0,1643	0,1159	1218,9813	0,7729
Russia	53	2425,5	2,919	1,1347	0,5333	1406,25	1312,50	1968,75	1,373	0,1185	0,0758	1250,2347	0,7393
Russia	53	2489,4	3,341	1,1593	0,5333	1406,25	1218,75	2062,50	1,334	0,1481	0,0972	1269,4293	0,7269
Russia	53	2553,2	3,160	1,2087	0,3093	1406,25	1312,50	2062,50	1,390	0,3156	0,0667	1287,1680	0,7333
Russia	53	2680,9	3,314	1,1593	0,5760	1500,00	1218,75	2156,25	1,492	0,1157	0,0602	1370,7947	0,7454
Russia	53	2553,2	3,412	1,2087	0,2773	1500,00	1312,50	2156,25	1,396	0,4336	0,1062	1402,7840	0,7699
Russia	53	2553,2	3,495	1,2827	0,6560	1500,00	1312,50	2156,25	1,408	0,1333	0,0958	1424,0533	0,7542
Russia	54	2224,4	3,017	1,0814	0,5227	1406,25	1312,50	1968,75	1,326	0,1095	0,0796	3283,6053	0,7562
Russia	54	2224,4	3,088	1,0640	0,5653	1406,25	1312,50	1968,75	1,261	0,0754	0,0553	3300,2027	0,7940
Russia	54	2165,9	3,319	1,0291	0,5547	1406,25	1218,75	2062,50	0,946	0,1414	0,0838	3311,0507	0,8429
Russia	54	2165,9	3,218	0,9767	0,6027	1406,25	1312,50	1968,75	1,457	0,0879	0,0440	3316,6720	0,8571
Russia	54	2165,9	3,512	0,9767	0,5600	1406,25	1125,00	2062,50	1,753	0,1093	0,0437	3320,4160	0,8306
Russia	54	2048,8	2,861	0,9419	0,3520	1312,50	1218,75	1781,25	1,293	0,2727	0,1023	3325,5680	0,7955
Russia	54	2224,4	3,458	0,9767	0,5387	1406,25	937,50	1968,75	1,015	0,1319	0,0549	3335,5413	0,8736
Russia	54	2165,9	3,338	1,1512	0,5813	1406,25	1218,75	1968,75	1,375	0,1023	0,0651	3390,0480	0,7721
Russia	54	2282,9	2,327	1,2384	0,2293	1312,50	1218,75	1500,00	1,321	0,3810	0,0736	3476,6560	0,6710
Russia	55	2042,6	2,752	0,9373	0,3627	1312,50	1125,00	1687,50	1,303	0,1494	0,1207	152,4480	0,7069
Russia	55	2297,9	3,466	0,8880	0,3680	1593,75	1312,50	2250,00	1,888	0,3494	0,0964	176,4107	0,8976
Russia	55	2489,4	3,585	1,1840	0,3413	1593,75	1312,50	2250,00	1,868	0,4480	0,1041	202,9387	0,8597
Russia	55	2170,2	2,825	1,0853	0,2613	1500,00	1312,50	1968,75	1,661	0,4532	0,0837	289,0187	0,8276
Russia	55	2234,0	2,614	1,0853	0,2667	1406,25	1218,75	1781,25	1,290	0,3960	0,0891	303,4347	0,7525
Russia	55	2234,0	2,509	1,0113	0,5227	1406,25	1218,75	1687,50	1,330	0,1481	0,0952	317,2427	0,8042

Russia	55	2361,7	2,939	1,1100	0,5013	1406,25	1218,75	1781,25	1,307	0,1490	0,1058	367,6747	0,7404
Russia	55	2297,9	2,557	1,0607	0,3147	1406,25	1312,50	1687,50	1,302	0,3401	0,0964	385,3600	0,7766
Russia	55	2489,4	2,818	1,0360	0,5653	1406,25	1218,75	1781,25	1,361	0,1192	0,0829	469,0080	0,7927
Russia	55	2234,0	2,884	1,1347	0,2453	1500,00	1312,50	1875,00	1,445	0,4387	0,1085	481,9147	0,7500
Finland	56	2202,9	3,105	0,8077	0,4053	1312,50	1125,00	1781,25	1,687	0,1589	0,0596	4,7413	0,8874
Finland	56	2434,8	3,245	0,8456	0,3893	1312,50	1125,00	1968,75	1,962	0,1266	0,0506	14,6453	0,8228
Finland	56	2144,9	3,156	0,9591	0,4907	1218,75	1031,25	1781,25	1,674	0,1676	0,0615	32,7787	0,9050
Finland	56	2087,0	2,996	0,9213	0,4320	1218,75	1031,25	1687,50	1,688	0,2035	0,0581	37,2640	0,8663
Finland	56	1855,1	2,976	0,9591	0,4267	1218,75	1031,25	1687,50	1,624	0,1955	0,0726	40,1653	0,8827
Finland	56	2144,9	2,867	0,8077	0,3307	1312,50	1125,00	1781,25	1,483	0,1333	0,0667	44,7360	0,8133
Finland	56	2144,9	2,811	0,9591	0,4480	1312,50	1125,00	1687,50	1,642	0,1517	0,0506	54,9013	0,8315
Finland	56	2202,9	3,075	0,8960	0,4213	1312,50	1125,00	1781,25	1,588	0,1377	0,0719	59,2533	0,8204
Finland	56	2144,9	3,065	0,9087	0,4320	1312,50	1125,00	1781,25	1,519	0,1361	0,0592	65,9893	0,8107
Finland	56	2260,9	3,010	0,8329	0,3947	1312,50	1125,00	1781,25	1,709	0,1419	0,0645	72,0000	0,8258
Finland	56	2144,9	2,983	0,9465	0,3893	1312,50	1125,00	1781,25	1,406	0,1356	0,0565	79,4293	0,7740
Finland	56	2202,9	3,041	0,8456	0,4213	1312,50	1125,00	1781,25	1,634	0,1592	0,0764	87,2000	0,8280
Finland	56	2029,0	2,646	0,9844	0,3733	1312,50	1125,00	1687,50	1,360	0,1304	0,0761	116,3253	0,7880
Finland	57	2608,7	3,571	0,9020	0,4427	1218,75	937,50	1968,75	1,539	0,1071	0,0417	7,8880	0,8571
Finland	57	2608,7	3,148	0,8224	0,3680	1312,50	1218,75	2062,50	1,421	0,2484	0,0458	12,2773	0,8889
Finland	57	2608,7	3,478	0,7428	0,3893	1312,50	1125,00	2062,50	1,496	0,1522	0,0652	14,7520	0,8986
Finland	57	2202,9	2,944	0,7588	0,2027	1406,25	1125,00	1875,00	1,419	0,4610	0,1206	21,9413	0,8865
Finland	57	2029,0	1,891	0,7269	0,4000	1218,75	1125,00	1406,25	1,254	0,1912	0,0809	29,8187	0,8382
Finland	58	2724,6	3,863	0,6739	0,2880	1312,50	1031,25	2250,00	1,764	0,1440	0,0800	6,4800	0,8240
Finland	58	2898,6	3,733	0,9216	0,5013	1218,75	937,50	2156,25	1,637	0,1345	0,0643	28,9973	0,8889
Finland	58	2898,6	3,756	0,8126	0,4053	1312,50	1031,25	2062,50	1,738	0,1184	0,0592	36,8587	0,8618
Finland	58	2550,7	3,688	0,7234	0,3200	1218,75	937,50	2062,50	1,543	0,2222	0,0963	41,3067	0,9037
Finland	58	2666,7	2,811	0,7433	0,1387	1406,25	1125,00	1875,00	1,400	0,5652	0,1232	52,6133	0,8986
Finland	58	2898,6	3,643	0,8225	0,3947	1125,00	843,75	1968,75	1,735	0,2222	0,0523	53,4347	0,9085
Finland	59	2550,7	3,669	0,7575	0,3467	1312,50	1031,25	2062,50	1,690	0,1631	0,1135	30,9653	0,8440
Finland	59	2492,8	3,790	0,7365	0,3893	1218,75	843,75	1968,75	1,557	0,1241	0,0730	36,0800	0,8394
Finland	59	2376,8	3,629	0,9048	0,4320	1312,50	1031,25	2062,50	1,775	0,1369	0,0833	58,8907	0,8690

Finland	59	2434,8	3,138	0,9679	0,3947	1218,75	1031,25	1875,00	1,428	0,1611	0,0833	64,2027	0,7556
Finland	59	2434,8	3,112	0,8417	0,4160	1312,50	1125,00	1875,00	1,471	0,1911	0,1083	102,1440	0,8854
Finland	59	2434,8	3,014	0,7996	0,3840	1218,75	1125,00	1875,00	1,381	0,1554	0,0743	109,7333	0,8446
Finland	59	2434,8	3,499	1,0311	0,4693	1312,50	1125,00	2062,50	1,448	0,2031	0,0885	132,8213	0,8333
Finland	60	2434,8	3,348	0,6940	0,3147	1125,00	843,75	1875,00	1,590	0,1473	0,0543	75,2107	0,8837
Finland	60	2376,8	3,510	0,6274	0,3040	1218,75	843,75	1875,00	1,488	0,1709	0,0598	80,7413	0,8803
Finland	60	2956,5	4,215	0,7329	0,4160	750,00	562,50	2062,50	2,235	0,1471	0,0368	88,8160	0,9191
Finland	60	2666,7	3,862	0,7218	0,3893	1125,00	843,75	2343,75	1,860	0,1791	0,0672	91,9360	0,9254
Finland	60	2840,6	4,016	0,7440	0,4373	1406,25	1031,25	2250,00	2,049	0,1667	0,0507	108,4320	0,9203
Finland	60	2782,6	3,996	0,6884	0,3733	1312,50	843,75	2250,00	1,897	0,1641	0,0469	126,6400	0,9141
Finland	60	2434,8	3,558	0,6551	0,3200	1593,75	1031,25	2156,25	1,903	0,1721	0,0574	170,9387	0,8525
Finland	60	2608,7	3,490	0,7162	0,4053	1312,50	1125,00	1968,75	1,825	0,1429	0,0602	181,5147	0,9248
Finland	60	2376,8	3,561	0,7273	0,3840	1312,50	937,50	1968,75	1,816	0,1259	0,0444	203,2587	0,8741
Finland	60	2318,8	3,221	0,6662	0,2133	1218,75	1031,25	1875,00	1,571	0,1371	0,0565	219,2800	0,8629
Finland	61	3188,4	3,323	1,2497	0,4267	1593,75	1406,25	2156,25	1,556	0,3991	0,0601	21,2640	0,8326
Finland	61	2782,6	3,390	1,1430	0,4107	1593,75	1312,50	2156,25	1,558	0,4299	0,0561	23,8027	0,8645
Finland	61	2840,6	3,762	0,9754	0,4213	1406,25	1218,75	2343,75	1,515	0,3901	0,0824	46,0160	0,8956
Finland	61	2956,5	3,126	1,0364	0,2080	1687,50	1593,75	2156,25	1,656	0,5130	0,1192	78,1227	0,8394
Finland	61	2492,8	2,855	0,9906	0,1867	1500,00	1406,25	1968,75	1,567	0,5784	0,1514	174,7627	0,8865
Finland	61	2202,9	3,073	1,1126	0,3947	1500,00	1312,50	1968,75	1,410	0,4203	0,0676	224,8533	0,8599
Finland	61	2666,7	2,912	1,0364	0,2560	1593,75	1500,00	2062,50	1,411	0,5309	0,0773	237,4507	0,8866
Finland	61	2492,8	3,229	1,2345	0,2720	1687,50	1500,00	2156,25	1,497	0,5844	0,4156	310,4267	0,8961
Finland	61	2724,6	3,625	1,1278	0,3733	1500,00	1312,50	2156,25	1,696	0,4882	0,0758	361,6693	0,9052
Finland	61	2608,7	3,181	0,9144	0,2453	1593,75	1406,25	2156,25	1,425	0,4176	0,1118	428,1387	0,8471
Finland	62	2608,7	2,805	1,1660	0,4000	1218,75	1125,00	2062,50	1,413	0,3670	0,0550	40,2027	0,9083
Finland	62	2550,7	2,461	1,0148	0,4587	1218,75	1125,00	1593,75	1,325	0,1316	0,0526	42,3947	0,8737
Finland	62	2376,8	2,640	1,2848	0,4213	1218,75	1125,00	1781,25	1,359	0,3473	0,0544	107,5360	0,8828
Finland	62	2434,8	2,720	1,1120	0,5547	1125,00	1125,00	1781,25	1,382	0,1442	0,0481	109,8133	0,8510
Finland	62	2492,8	2,297	1,1444	0,2560	1218,75	1125,00	1687,50	1,287	0,3832	0,0981	145,2587	0,8738
Finland	62	2550,7	2,688	1,0041	0,2880	1218,75	1125,00	1781,25	1,341	0,3262	0,0749	149,0133	0,8877
Finland	62	3072,5	2,670	1,0148	0,2027	1218,75	1125,00	1875,00	1,351	0,3947	0,0947	216,4053	0,8368

Finland	62	2840,6	2,362	1,1228	0,3413	1218,75	1125,00	1687,50	1,332	0,2679	0,0718	259,2960	0,8756
Finland	62	2550,7	2,328	1,2740	0,2933	1218,75	1125,00	1781,25	1,301	0,3235	0,0714	303,2853	0,8655
Finland	63	2376,8	2,405	0,9349	0,1600	1500,00	1406,25	1781,25	1,282	0,5805	0,0920	25,6907	0,8736
Finland	63	2898,6	2,965	0,8681	0,1600	1500,00	1312,50	1968,75	1,395	0,5652	0,1801	41,8453	0,8634
Finland	63	2492,8	2,507	0,9516	0,1707	1500,00	1406,25	1875,00	1,382	0,5367	0,0678	70,4267	0,8362
Finland	63	2550,7	2,747	0,7680	0,4160	1500,00	1312,50	1875,00	1,431	0,2028	0,0769	72,3840	0,8671
Finland	63	2376,8	2,769	0,8180	0,1547	1500,00	1406,25	1968,75	1,400	0,4868	0,1118	86,3627	0,8092
Finland	63	2260,9	2,587	0,7346	0,3573	1500,00	1312,50	1875,00	1,349	0,1460	0,0876	88,5920	0,7445
Finland	64	2434,8	3,317	1,0916	0,3200	1406,25	1125,00	1968,75	1,472	0,4510	0,0833	31,2693	0,8333
Finland	64	2318,8	3,202	1,0656	0,2987	1500,00	1218,75	1875,00	1,431	0,4646	0,0808	133,0613	0,8182
Finland	64	2492,8	3,195	0,9487	0,5387	1406,25	1218,75	1875,00	1,530	0,1017	0,0791	139,8827	0,7797
Finland	64	2434,8	3,242	0,9877	0,4587	1406,25	1125,00	1875,00	1,388	0,2989	0,0924	147,6160	0,8696
Finland	64	2434,8	3,122	1,0396	0,2720	1593,75	1218,75	1968,75	1,571	0,5309	0,0876	187,0080	0,8557
Finland	64	2376,8	3,558	1,0786	0,5013	1218,75	1125,00	1968,75	1,317	0,1642	0,0796	250,6133	0,8109
Finland	64	2202,9	3,359	1,0136	0,3733	1312,50	1125,00	1875,00	1,664	0,3810	0,1058	253,5840	0,8571
Finland	64	2492,8	3,105	1,1046	0,2773	1500,00	1218,75	1875,00	1,455	0,4660	0,1068	293,0880	0,7816
Finland	64	2318,8	3,295	1,0396	0,3947	1406,25	1125,00	1968,75	1,565	0,3660	0,0876	299,7067	0,8196
Finland	64	2492,8	3,201	0,9357	0,3680	1500,00	1218,75	1875,00	1,573	0,3429	0,0800	308,6880	0,8229
Finland	64	2376,8	3,408	0,7277	0,3893	1312,50	1125,00	1875,00	1,592	0,1778	0,0889	318,4640	0,8963
Finland	64	2376,8	3,369	1,0396	0,5600	1312,50	1125,00	1875,00	1,655	0,1701	0,0722	348,2560	0,8299
Finland	65	2724,6	3,133	1,0884	0,2027	1593,75	1312,50	2062,50	1,522	0,5517	0,0739	18,8960	0,8128
Finland	65	2492,8	3,451	0,8985	0,2347	1593,75	1218,75	2062,50	1,615	0,5536	0,1012	22,4587	0,8929
Finland	65	2608,7	3,269	1,2782	0,2667	1593,75	1312,50	2062,50	1,535	0,5188	0,1088	59,9147	0,8033
Finland	65	2492,8	3,463	0,8606	0,4800	1406,25	1218,75	1968,75	1,600	0,2188	0,1000	74,2240	0,8562
Finland	65	2434,8	3,501	0,7846	0,4533	1406,25	1218,75	2062,50	1,574	0,2260	0,0753	81,0187	0,8904
Finland	66	2550,7	3,379	1,2532	0,3040	1312,50	1218,75	1968,75	1,664	0,3248	0,0470	5,5573	0,7393
Finland	66	2318,8	2,893	1,1072	0,2507	1593,75	1218,75	1875,00	1,345	0,5291	0,1311	22,3040	0,8107
Finland	66	2492,8	3,195	0,9612	0,3040	1500,00	1218,75	1968,75	1,606	0,5140	0,0950	92,3840	0,8827
Finland	66	2550,7	3,149	0,8882	0,3253	1500,00	1218,75	1968,75	1,509	0,4639	0,0783	113,0187	0,8916
Finland	66	2492,8	3,208	0,8517	0,4693	1312,50	1125,00	1875,00	1,474	0,1698	0,0692	119,0400	0,8239
Finland	66	2376,8	2,757	0,8761	0,2240	1593,75	1218,75	1875,00	1,261	0,5793	0,0976	127,6587	0,8780

Finland	66	2492,8	2,873	0,9856	0,2240	1500,00	1218,75	1875,00	1,515	0,5761	0,0924	141,8347	0,8750
Finland	66	2608,7	3,459	0,9491	0,5547	1406,25	1125,00	1968,75	1,763	0,1307	0,0625	162,9120	0,8580
Finland	66	2666,7	3,507	0,9004	0,5440	1406,25	1125,00	1968,75	1,535	0,1905	0,0655	172,8853	0,8929
Finland	66	2608,7	3,492	0,8396	0,4907	1312,50	1125,00	1968,75	1,520	0,1795	0,0897	176,6773	0,8846
Finland	67	2608,7	3,446	0,8162	0,2187	1593,75	1218,75	2156,25	1,660	0,5461	0,0789	103,2800	0,8816
Finland	67	2260,9	3,360	0,9482	0,5867	1312,50	1125,00	1968,75	1,522	0,0909	0,0568	173,3013	0,8693
Finland	67	2376,8	3,320	0,8071	0,5013	1406,25	1125,00	1968,75	1,513	0,1060	0,0662	193,4507	0,8675
Finland	67	2492,8	3,247	0,8762	0,6133	1406,25	1125,00	1968,75	1,496	0,0798	0,0491	212,5440	0,8589
Finland	67	2318,8	3,223	0,6122	0,3093	1218,75	1031,25	1781,25	1,615	0,1579	0,0702	217,5200	0,8509
Finland	67	2434,8	3,261	0,8738	0,3733	1406,25	1218,75	1968,75	1,743	0,3558	0,0798	18,1333	0,9018
Finland	67	2550,7	3,309	0,9198	0,4693	1500,00	1218,75	1968,75	1,486	0,2733	0,0640	51,7600	0,8547
Finland	67	2376,8	3,436	0,8585	0,5067	1406,25	1125,00	1968,75	1,540	0,2062	0,0938	65,3173	0,8688
Finland	67	2144,9	3,289	0,6439	0,3840	1406,25	1218,75	1968,75	1,769	0,1849	0,0924	72,8853	0,8824
Finland	68	2318,8	2,517	1,0152	0,5067	1406,25	1218,75	1781,25	1,297	0,1105	0,0684	21,6480	0,7684
Finland	68	2434,8	2,824	1,0383	0,4533	1406,25	1312,50	1875,00	1,426	0,0984	0,0725	45,4293	0,7409
Finland	68	2202,9	2,735	1,0383	0,5120	1312,50	1218,75	1875,00	1,332	0,1082	0,0825	77,6907	0,7990
Finland	68	2202,9	2,459	0,9921	0,3893	1406,25	1312,50	1687,50	1,329	0,1189	0,0865	128,8693	0,7892
Finland	68	2202,9	2,479	0,9460	0,4640	1312,50	1218,75	1781,25	1,340	0,0966	0,0682	143,2533	0,8352
Finland	68	2202,9	2,716	0,9921	0,4320	1312,50	1218,75	1875,00	1,324	0,1189	0,0865	163,9040	0,7459
Finland	68	2318,8	2,353	0,9690	0,4907	1312,50	1218,75	1781,25	1,321	0,1050	0,0773	277,7973	0,7956
Finland	68	2434,8	2,714	0,9921	0,4427	1406,25	1218,75	1875,00	1,416	0,1081	0,0811	302,2613	0,8054
Finland	68	2434,8	2,688	0,9921	0,4587	1312,50	1312,50	1875,00	1,285	0,1297	0,1027	409,8507	0,8162
Finland	69	3072,5	3,603	0,6614	0,3893	1687,50	1500,00	2343,75	1,455	0,1870	0,0813	19,4667	0,8699
Finland	69	2898,6	3,299	0,8475	0,1920	1593,75	1406,25	2156,25	1,596	0,5949	0,0696	54,3307	0,8861
Finland	69	2782,6	3,471	0,7478	0,4533	1500,00	1312,50	2062,50	1,914	0,1655	0,0576	3,0987	0,8561
Finland	69	2724,6	3,502	0,7818	0,2027	1593,75	1312,50	2156,25	1,887	0,5655	0,0690	25,3867	0,9103
Finland	69	2724,6	3,147	0,8611	0,2027	1687,50	1500,00	2156,25	1,638	0,5963	0,0807	35,8880	0,8944
Finland	69	2608,7	3,193	0,6685	0,3733	1593,75	1406,25	2062,50	1,762	0,1040	0,0640	59,5893	0,8240
Finland	70	2260,9	3,089	0,9971	0,4587	1312,50	1218,75	1968,75	1,425	0,1129	0,0645	60,1653	0,8172
Finland	70	2202,9	2,534	0,7994	0,4533	1312,50	1218,75	1781,25	1,335	0,1007	0,0604	64,9333	0,8792
Finland	70	2550,7	2,497	0,8044	0,2720	1406,25	1312,50	1875,00	1,396	0,1600	0,1000	29,0187	0,7467

Finland	70	2144,9	2,634	1,0424	0,5493	1406,25	1218,75	1781,25	1,321	0,0773	0,0464	149,7387	0,8196
Finland	70	2376,8	3,037	0,9177	0,4853	1312,50	1218,75	1968,75	1,382	0,1287	0,0760	156,0480	0,8480
Finland	70	2144,9	2,471	0,8611	0,4427	1312,50	1218,75	1687,50	1,377	0,1062	0,0688	231,7387	0,8375
Finland	71	2029,0	2,477	0,9779	0,4960	1312,50	1218,75	1687,50	1,265	0,1421	0,0546	0,9600	0,8251
Finland	71	2144,9	2,319	0,8363	0,4480	1312,50	1218,75	1593,75	1,261	0,1218	0,0449	6,9867	0,8013
Finland	71	2260,9	2,724	0,8492	0,4213	1312,50	1218,75	1687,50	1,361	0,1456	0,0759	14,2987	0,8418
Finland	71	2087,0	2,339	0,9521	0,4053	1312,50	1218,75	1593,75	1,302	0,1582	0,0847	185,1093	0,7514
Finland	71	2376,8	2,466	0,9521	0,4373	1312,50	1218,75	1593,75	1,255	0,1412	0,0565	197,0560	0,7288
Finland	71	2376,8	2,475	0,9135	0,4000	1218,75	1125,00	1593,75	1,301	0,1520	0,0819	219,6960	0,7661
Finland	71	2550,7	2,581	1,0036	0,4107	1312,50	1218,75	1687,50	1,294	0,1123	0,0535	306,8160	0,6952
Finland	71	2376,8	2,551	0,9521	0,4160	1312,50	1218,75	1593,75	1,435	0,1180	0,0562	313,0507	0,7528
Finland	71	2202,9	2,468	0,9521	0,4480	1312,50	1218,75	1593,75	1,276	0,1243	0,0734	321,9893	0,7514
Finland	71	2144,9	2,395	0,9007	0,3200	1312,50	1218,75	1593,75	1,275	0,1131	0,0595	343,7173	0,7143
Finland	71	2318,8	2,581	0,9907	0,5653	1312,50	1218,75	1593,75	1,325	0,0973	0,0595	366,5067	0,8324
Finland	71	2376,8	2,458	0,9907	0,4853	1312,50	1218,75	1593,75	1,299	0,1243	0,0919	392,5013	0,7838
Finland	71	2087,0	2,677	0,8878	0,4053	1312,50	1125,00	1593,75	1,276	0,1386	0,0663	490,6027	0,7831
Finland	72	2434,8	3,112	1,1358	0,3147	1406,25	1312,50	1968,75	1,537	0,5142	0,0991	1,5413	0,8679
Finland	72	2376,8	3,048	1,0985	0,6133	1312,50	1218,75	1875,00	1,633	0,0927	0,0537	51,3973	0,8244
Finland	72	2260,9	3,179	1,1358	0,6187	1218,75	1125,00	1781,25	1,333	0,1321	0,0896	58,6027	0,7642
Finland	72	2492,8	3,137	1,1730	0,2613	1406,25	1312,50	1968,75	1,624	0,4886	0,0822	72,4747	0,8447
Finland	72	2318,8	3,034	1,0613	0,6293	1406,25	1218,75	1875,00	1,363	0,0707	0,0404	77,7227	0,7879
Finland	72	2434,8	3,117	1,0799	0,1973	1593,75	1125,00	1968,75	1,546	0,4975	0,0995	130,3413	0,8109
Finland	73	2029,0	2,175	1,0848	0,3733	1218,75	1125,00	1500,00	1,263	0,1921	0,1232	54,6293	0,7291
Finland	73	1855,1	2,101	1,0286	0,3093	1218,75	1125,00	1500,00	1,237	0,1728	0,1099	84,4800	0,6963
Finland	73	1971,0	2,198	0,9164	0,3253	1218,75	1125,00	1406,25	1,303	0,1988	0,1345	94,5280	0,7427
Finland	73	1855,1	2,369	1,0099	0,3253	1218,75	1031,25	1406,25	1,336	0,2287	0,1117	115,9893	0,7553
Finland	73	1971,0	2,220	0,9725	0,3573	1218,75	1125,00	1500,00	1,280	0,1768	0,1105	133,2000	0,7238
Finland	73	2087,0	2,307	1,0286	0,4267	1218,75	1125,00	1500,00	1,324	0,1562	0,0938	148,0427	0,7448
Finland	73	2087,0	2,112	1,0661	0,3253	1218,75	1125,00	1500,00	1,284	0,1717	0,1010	206,1067	0,6818
Finland	73	2087,0	2,391	0,9351	0,4267	1218,75	1125,00	1593,75	1,341	0,1897	0,1092	241,7333	0,8333
Finland	74	2608,7	3,565	0,7835	0,5013	1593,75	1218,75	2250,00	1,906	0,1233	0,0548	17,8400	0,8699

Finland	74	3014,5	4,063	0,7664	0,3040	1500,00	1218,75	2625,00	1,927	0,4085	0,1056	38,1387	0,9225
Finland	74	3130,4	3,932	0,8005	0,4747	1500,00	1218,75	2531,25	1,780	0,1409	0,0872	167,0133	0,8725
Finland	74	2782,6	3,869	0,7835	0,3733	1406,25	1218,75	2343,75	1,737	0,2603	0,1027	171,4987	0,8356
Finland	74	2898,6	3,059	0,7664	0,4053	1593,75	1312,50	2156,25	1,485	0,0979	0,0699	194,4960	0,8182
Finland	74	2724,6	3,333	1,0327	0,1973	1687,50	1406,25	2156,25	1,859	0,5521	0,0677	30,1760	0,8490
Finland	74	2782,6	3,306	1,0014	0,2560	1593,75	1406,25	2156,25	1,716	0,4706	0,0642	50,7733	0,8396
Finland	74	3014,5	3,983	0,9957	0,2613	1687,50	1406,25	2625,00	1,846	0,4839	0,0753	113,0187	0,8441
Finland	75	2290,2	2,636	1,1005	0,3193	1378,13	1291,99	1722,66	1,516	0,4762	0,0423	1,0797	0,8677
Finland	75	2023,9	2,673	1,1727	0,5921	1378,13	1205,86	1722,66	1,490	0,1891	0,0398	6,8150	0,8607
Finland	75	2023,9	2,539	1,0284	0,5863	1378,13	1205,86	1636,52	1,395	0,1412	0,0621	10,5651	0,8701
Finland	75	2130,4	2,706	1,1186	0,3251	1378,13	1291,99	1722,66	1,303	0,4271	0,0625	18,9126	0,8698
Finland	75	2077,2	2,582	1,0825	0,6037	1378,13	1291,99	1636,52	1,454	0,1243	0,0486	38,9224	0,8541
Finland	75	2290,2	2,557	1,0645	0,5050	1291,99	1205,86	1636,52	1,292	0,1257	0,0656	44,3385	0,8197
Finland	75	2396,7	2,874	1,1547	0,7198	1378,13	1291,99	1808,79	1,418	0,1768	0,0758	63,3789	0,9040
Finland	75	2237,0	2,858	1,1005	0,5515	1291,99	1205,86	1722,66	1,491	0,1005	0,0423	68,2086	0,8307
Finland	75	2237,0	2,853	1,1186	0,3077	1378,13	1205,86	1808,79	1,360	0,4167	0,0990	86,8716	0,8698
Finland	75	2290,2	2,674	1,0825	0,5457	1291,99	1205,86	1722,66	1,438	0,1027	0,0541	93,7157	0,8378
Finland	75	2237,0	3,036	1,1186	0,3715	1378,13	1205,86	1808,79	1,667	0,3927	0,0733	119,1822	0,8796
Finland	75	2130,4	2,584	1,1727	0,5805	1378,13	1291,99	1636,52	1,389	0,1542	0,0647	130,5019	0,8358
Finland	75	2237,0	2,784	1,1727	0,7082	1378,13	1291,99	1722,66	1,539	0,1095	0,0597	149,0547	0,8706
Finland	75	2343,5	2,920	1,2088	0,5573	1378,13	1291,99	1808,79	1,564	0,1208	0,0628	153,8844	0,8551
Finland	75	2183,7	2,964	1,1727	0,6502	1378,13	1291,99	1894,92	1,547	0,0945	0,0498	193,7299	0,8507
Finland	75	2183,7	2,825	1,2268	0,6037	1291,99	1205,86	1722,66	1,497	0,1524	0,0762	213,8035	0,8619
Finland	75	2343,5	2,622	1,1727	0,2670	1378,13	1205,86	1722,66	1,485	0,5025	0,1244	229,0706	0,8756
Finland	75	2237,0	2,674	1,1908	0,6850	1378,13	1291,99	1722,66	1,413	0,1324	0,0588	250,5201	0,8480
Finland	76	2450,0	3,087	0,8568	0,3657	1119,73	947,46	1808,79	1,633	0,1769	0,1156	13,0148	0,8639
Finland	76	2450,0	3,271	0,7153	0,2554	1205,86	947,46	1722,66	1,568	0,2049	0,0656	57,2488	0,8607
Finland	76	2343,5	2,810	0,9983	0,3831	1119,73	1033,59	1722,66	1,370	0,1228	0,0409	103,2823	0,8363
Finland	76	2183,7	2,556	0,9118	0,3773	1119,73	1033,59	1464,26	1,449	0,1795	0,0577	116,3784	0,7885
Finland	76	2130,4	2,856	0,8096	0,3657	1119,73	861,33	1550,39	1,631	0,1232	0,0507	119,5653	0,8768
Finland	76	2290,2	2,377	0,9590	0,4180	1205,86	1033,59	1378,13	1,408	0,1463	0,0549	146,7907	0,8049

Finland	76	2290,2	2,928	1,0533	0,4470	1205,86	1119,73	1722,66	1,524	0,1823	0,0442	231,7177	0,8950
Finland	77	2237,0	3,842	1,0690	0,4470	1205,86	861,33	1981,06	1,837	0,4076	0,0598	92,4096	0,9402
Finland	77	2556,5	4,046	1,0847	0,6269	1378,13	1033,59	2239,45	2,193	0,1882	0,0376	108,3907	0,9140
Finland	77	2450,0	3,661	0,9197	0,4760	1464,26	1205,86	2153,32	1,595	0,2102	0,0127	111,4035	0,9299
Finland	77	2503,3	3,611	1,1083	0,3193	1550,39	1205,86	2153,32	1,592	0,4947	0,0842	123,2457	0,9211
Finland	77	2769,6	3,646	1,1005	0,5050	1464,26	1205,86	2067,19	1,663	0,4149	0,0426	139,1049	0,9415
Finland	77	2769,6	3,448	1,1555	0,3309	1550,39	1378,13	2153,32	1,425	0,5606	0,3737	191,4891	0,9343
Finland	77	2556,5	3,377	1,1162	0,3773	1464,26	1291,99	2153,32	1,438	0,4503	0,0419	273,0086	0,9215
Finland	77	2450,0	3,476	1,2184	0,4063	1550,39	1205,86	2067,19	1,606	0,4306	0,1148	295,6481	0,8708
Finland	78	2343,5	3,675	0,9221	0,3425	1550,39	1205,86	2153,32	2,217	0,4684	0,1139	24,5028	0,9241
Finland	78	2556,5	3,966	1,0091	0,5050	1464,26	1205,86	2325,59	1,920	0,3023	0,0523	34,1449	0,9012
Finland	78	2663,0	3,937	0,8873	0,3599	1464,26	1291,99	2411,72	2,040	0,3487	0,1053	60,4531	0,8750
Finland	78	2556,5	3,786	0,9395	0,3889	1550,39	1205,86	2239,45	1,818	0,3727	0,1056	78,4428	0,8882
Finland	78	2503,3	3,655	0,6959	0,3309	1464,26	1205,86	2153,32	2,027	0,2941	0,0588	85,3101	0,9076
Finland	78	2609,8	3,596	0,8525	0,4528	1550,39	1378,13	2239,45	1,730	0,1781	0,0822	110,4399	0,8630
Finland	78	2876,1	4,022	0,9395	0,3947	1464,26	1205,86	2583,98	1,967	0,3354	0,0932	188,6389	0,8758
Finland	79	2237,0	3,247	0,9569	0,5399	1378,13	1119,73	1894,92	1,519	0,1840	0,0920	29,6983	0,8589
Finland	79	2237,0	3,636	1,0786	0,4180	1291,99	1119,73	1981,06	1,508	0,3243	0,0703	59,2341	0,8486
Finland	79	2183,7	2,807	0,9917	0,1916	1550,39	1291,99	1808,79	1,489	0,5471	0,0941	140,3530	0,8353
Finland	79	2130,4	2,873	0,8351	0,2148	1464,26	1291,99	1808,79	1,351	0,4825	0,0699	143,0059	0,8392
Finland	79	2237,0	3,272	0,9221	0,2961	1464,26	1205,86	1894,92	1,626	0,4747	0,0759	180,9241	0,8797
Finland	79	2290,2	3,170	0,8003	0,3773	1291,99	1205,86	1808,79	1,596	0,1985	0,0662	183,1126	0,8162
Finland	79	2396,7	3,283	0,9047	0,2380	1464,26	1205,86	1894,92	1,544	0,4710	0,1097	227,9735	0,8323
Finland	79	2237,0	3,261	0,8525	0,4528	1378,13	1205,86	1894,92	1,681	0,1575	0,0822	232,4143	0,8493
Finland	80	2183,7	2,741	0,6656	0,3715	1464,26	1291,99	1808,79	1,317	0,0877	0,0526	11,5055	0,7719
Finland	80	2130,4	2,304	0,6656	0,3889	1464,26	1378,13	1722,66	1,254	0,1062	0,0796	27,1267	0,8407
Finland	80	2237,0	2,691	0,6798	0,4238	1550,39	1378,13	1808,79	1,346	0,1034	0,0690	35,7703	0,8621
Finland	80	2077,2	2,483	0,6939	0,4063	1464,26	1378,13	1808,79	1,267	0,1176	0,0756	57,1559	0,8235
Finland	80	2130,4	2,769	0,6231	0,3251	1464,26	1291,99	1808,79	1,494	0,1215	0,0748	59,8668	0,8131
Finland	80	2023,9	1,942	0,6089	0,0987	1378,13	1291,99	1550,39	1,288	0,1154	0,0673	73,2183	0,7692
Finland	80	2023,9	2,782	0,6231	0,3541	1378,13	1291,99	1808,79	1,437	0,1226	0,0660	78,5183	0,8491

Finland	80	2130,4	2,681	0,6798	0,3483	1378,13	1291,99	1808,79	1,296	0,1379	0,0690	98,4700	0,8190
Finland	80	2077,2	2,475	0,7789	0,4180	1464,26	1378,13	1722,66	1,239	0,1203	0,0902	141,0322	0,8195
Finland	81	2130,4	3,089	0,8919	0,4470	1378,13	1119,73	1722,66	1,478	0,1382	0,0921	18,3089	0,7829
Finland	81	2077,2	2,832	0,8540	0,4470	1464,26	1291,99	1722,66	1,319	0,1233	0,0753	54,5321	0,7534
Finland	81	2130,4	2,523	0,8730	0,4238	1464,26	1291,99	1722,66	1,318	0,1467	0,0933	123,7856	0,7200
Finland	81	2609,8	2,342	1,1007	0,5805	1550,39	1464,26	1808,79	1,435	0,0794	0,0582	204,6084	0,7143
Finland	81	2450,0	2,740	1,0248	0,5515	1464,26	1378,13	1808,79	1,399	0,0795	0,0568	236,5417	0,7386
Finland	82	2077,2	2,931	1,0106	0,3831	1291,99	1119,73	1722,66	1,538	0,1792	0,0520	12,9625	0,7514
Finland	82	1970,7	2,380	0,8141	0,2612	1205,86	1205,86	1550,39	1,347	0,1942	0,0791	15,7838	0,7986
Finland	82	2183,7	2,700	0,9966	0,3657	1205,86	1119,73	1636,52	1,358	0,2105	0,0994	51,7457	0,8012
Finland	82	2077,2	2,382	1,0106	0,4005	1291,99	1119,73	1550,39	1,248	0,1561	0,0520	59,4721	0,8497
Finland	82	1970,7	2,232	0,7580	0,2612	1205,86	1119,73	1464,26	1,307	0,1615	0,0538	62,2817	0,7846
Finland	82	1970,7	2,581	0,9124	0,2961	1205,86	1119,73	1550,39	1,392	0,2051	0,0897	66,3220	0,7885
Finland	82	1917,4	2,419	1,0106	0,3831	1205,86	1119,73	1550,39	1,344	0,1437	0,0747	69,7411	0,7586
Finland	82	2183,7	2,433	1,0247	0,2844	1291,99	1119,73	1550,39	1,428	0,2102	0,0568	94,0234	0,7045
Finland	82	2023,9	2,226	0,7720	0,2844	1205,86	1119,73	1464,26	1,286	0,2121	0,0682	97,8315	0,7955
Finland	82	2077,2	2,438	1,0668	0,4180	1205,86	1205,86	1550,39	1,286	0,1749	0,0929	101,0765	0,8142
Finland	82	1917,4	2,184	0,8983	0,3425	1205,86	1119,73	1464,26	1,350	0,2078	0,0844	104,9484	0,8377
Finland	82	2023,9	2,349	0,8703	0,2961	1205,86	1205,86	1550,39	1,317	0,2215	0,0940	113,8881	0,8054
Finland	82	1864,1	1,994	0,8983	0,3135	1205,86	1205,86	1464,26	1,296	0,1307	0,0654	117,0866	0,7190
Finland	82	2077,2	2,545	1,0949	0,3077	1291,99	1205,86	1636,52	1,397	0,1968	0,0691	129,7821	0,6809
Finland	82	2077,2	2,589	0,9545	0,3367	1291,99	1205,86	1636,52	1,353	0,1534	0,0613	137,4679	0,7178
Finland	82	2077,2	2,689	1,0247	0,3657	1291,99	1119,73	1636,52	1,439	0,1771	0,0800	144,8228	0,7771
Finland	82	1970,7	2,234	1,0387	0,3831	1291,99	1205,86	1550,39	1,285	0,1573	0,0730	149,5539	0,7303
Finland	82	2077,2	2,122	1,1229	0,3715	1291,99	1205,86	1550,39	1,253	0,1865	0,0881	155,5621	0,7565
Finland	82	1970,7	2,072	0,9405	0,2902	1205,86	1119,73	1464,26	1,252	0,1739	0,0745	157,9711	0,6894
Finland	82	2183,7	2,422	0,9405	0,3135	1291,99	1205,86	1636,52	1,302	0,1739	0,0932	162,3655	0,7516
Finland	82	2130,4	2,608	0,8562	0,2322	1291,99	1205,86	1636,52	1,348	0,3562	0,1301	168,1531	0,8356
Finland	82	2077,2	2,523	0,8983	0,2438	1291,99	1205,86	1636,52	1,375	0,3182	0,1039	176,5355	0,8117
Finland	82	2077,2	2,100	0,9966	0,3251	1291,99	1205,86	1550,39	1,291	0,1754	0,0936	180,5526	0,7602
Finland	82	2130,4	2,347	0,9124	0,2728	1291,99	1205,86	1636,52	1,209	0,1667	0,0833	187,1702	0,7051

Finland	82	2199,6	2,782	0,8703	0,3309	1291,99	1205,86	1722,66	1,437	0,2215	0,1007	196,3711	0,8456
Finland	82	2038,7	2,189	1,0247	0,3947	1291,99	1205,86	1550,39	1,277	0,1143	0,0571	200,8758	0,7429

Tabele S1: Values of the variables included in the resulting DFA model.
